# The interplays between Crimean-Congo hemorrhagic fever virus (CCHFV) M segment-encoded accessory proteins and structural proteins promote virus assembly and infectivity

**DOI:** 10.1371/journal.ppat.1008850

**Published:** 2020-09-21

**Authors:** Natalia Freitas, Margot Enguehard, Solène Denolly, Camille Levy, Gregory Neveu, Solène Lerolle, Stephanie Devignot, Friedemann Weber, Eric Bergeron, Vincent Legros, François-Loïc Cosset

**Affiliations:** 1 CIRI–Centre International de Recherche en Infectiologie, Univ Lyon, Université Claude Bernard Lyon 1, Inserm, U1111, CNRS, UMR5308, ENS Lyon, 46 allée d’Italie, Lyon, France; 2 Institute for Virology, FB10-Veterinary Medicine, Justus-Liebig University, Gießen, Germany; 3 Viral Special Pathogens Branch, Division of High-Consequence Pathogens and Pathology, Centers for Disease Control and Prevention, Atlanta, Georgia, United States of America; 4 Université de Lyon, VetAgro Sup, Marcy-l'Étoile, France; Division of Clinical Research, UNITED STATES

## Abstract

Crimean-Congo hemorrhagic fever virus (CCHFV) is a tick-borne *orthonairovirus* that has become a serious threat to the public health. CCHFV has a single-stranded, tripartite RNA genome composed of L, M, and S segments. Cleavage of the M polyprotein precursor generates the two envelope glycoproteins (GPs) as well as three secreted nonstructural proteins GP38 and GP85 or GP160, representing GP38 only or GP38 linked to a mucin-like protein (MLD), and a double-membrane-spanning protein called NSm. Here, we examined the relevance of each M-segment non-structural proteins in virus assembly, egress and infectivity using a well-established CCHFV virus-like-particle system (tc-VLP). Deletion of MLD protein had no impact on infectivity although it reduced by 60% incorporation of GPs into particles. Additional deletion of GP38 abolished production of infectious tc-VLPs. The loss of infectivity was associated with impaired Gc maturation and exclusion from the Golgi, showing that Gn is not sufficient to target CCHFV GPs to the site of assembly. Consistent with this, efficient complementation was achieved in cells expressing MLD-GP38 *in trans* with increased levels of preGc to Gc conversion, co-targeting to the Golgi, resulting in particle incorporation and restored infectivity. Contrastingly, a MLD-GP38 variant retained in the ER allowed preGc cleavage but failed to rescue miss-localization or infectivity. NSm deletion, conversely, did not affect trafficking of Gc but interfered with Gc processing, particle formation and secretion. NSm expression affected N-glycosylation of different viral proteins most likely due to increased speed of trafficking through the secretory pathway. This highlights a potential role of NSm in overcoming Golgi retention and facilitating CCHFV egress. Thus, deletions of GP38 or NSm demonstrate their important role on CCHFV particle production and infectivity. GP85 is an essential viral factor for preGc cleavage, trafficking and Gc incorporation into particles, whereas NSm protein is involved in CCHFV assembly and virion secretion.

## Author summary

*Orthonairoviruses*, like the lethal Crimean-Congo hemorrhagic fever virus (CCHFV), encode secreted glycoproteins, such as GP38, in addition to virion envelope glycoproteins (Gn and Gc) that are processed by internal cleavage of the viral M segment encoded polyprotein. CCHFV MLD-GP38 proteins (GP160/GP85) also include an N-terminal domain encompassing a mucin-like protein that is released from GP38 by Furin. The protective effect of non-neutralizing monoclonal antibodies targeting GP38 against lethal CCHFV challenge previously highlighted the importance of GP38 in CCHFV replication. CCHFV also encodes a double-membrane-spanning protein (NSm) of unknown function, located between the Gn and Gc on the polyprotein. To investigate the roles of these so-called accessory proteins encoded by the CCHFV M-segment in virus formation and infectivity, we generated several M-segment deletion mutants and tested them in a CCHFV transcription-entry-competent virus-like particle (tc-VLP) system. Here, we demonstrate that GP38 is crucial for Gc biogenesis, interaction with Gn and trafficking to the Golgi, and that its deletion abrogates formation of infectious particles. We also show that NSm increases the rate of protein trafficking through the secretory pathway with altered N-glycosylation profiles that are advantageous for efficient virus release. These data advanced our understanding of GP38 and NSm roles and CCHFV-host interactions.

## Introduction

The genus *Orthonairovirus* belongs to the family *Nairoviridae* of the order *Bunyavirales* and consists of at least 43 viruses assigned to 15 groups/species [1]. They are tick-borne viruses that circulate in nature in enzootic cycles involving ticks and vertebrates that can act as amplifying hosts [2–4]. Ticks are considered as both the vector and the main reservoir, and transmit the virus when feeding on animals [5]. The genus is named after the Nairobi sheep disease virus (NSDV), responsible for often-fatal acute hemorrhagic gastroenteritis in sheep and goats. Its most significant human pathogen, is the Crimean-Congo hemorrhagic fever virus (CCHFV), producing a severe hemorrhagic disease with a mortality rate of up to 30%. Because of its epidemic potential, the high rate of mortality, the possibility of nosocomial infections, and lack of specific treatment options and preventive strategies, CCHFV poses a serious threat to the world health [6]. CCHFV activity closely mirrors the geographic distribution of its vectors, and since 2000, the incidence and range of confirmed CCHF cases have markedly increased, probably due to the geographical expansion of Hyalomma spp. ticks, an important tick vector of CCHFV.

**Fig 1:**
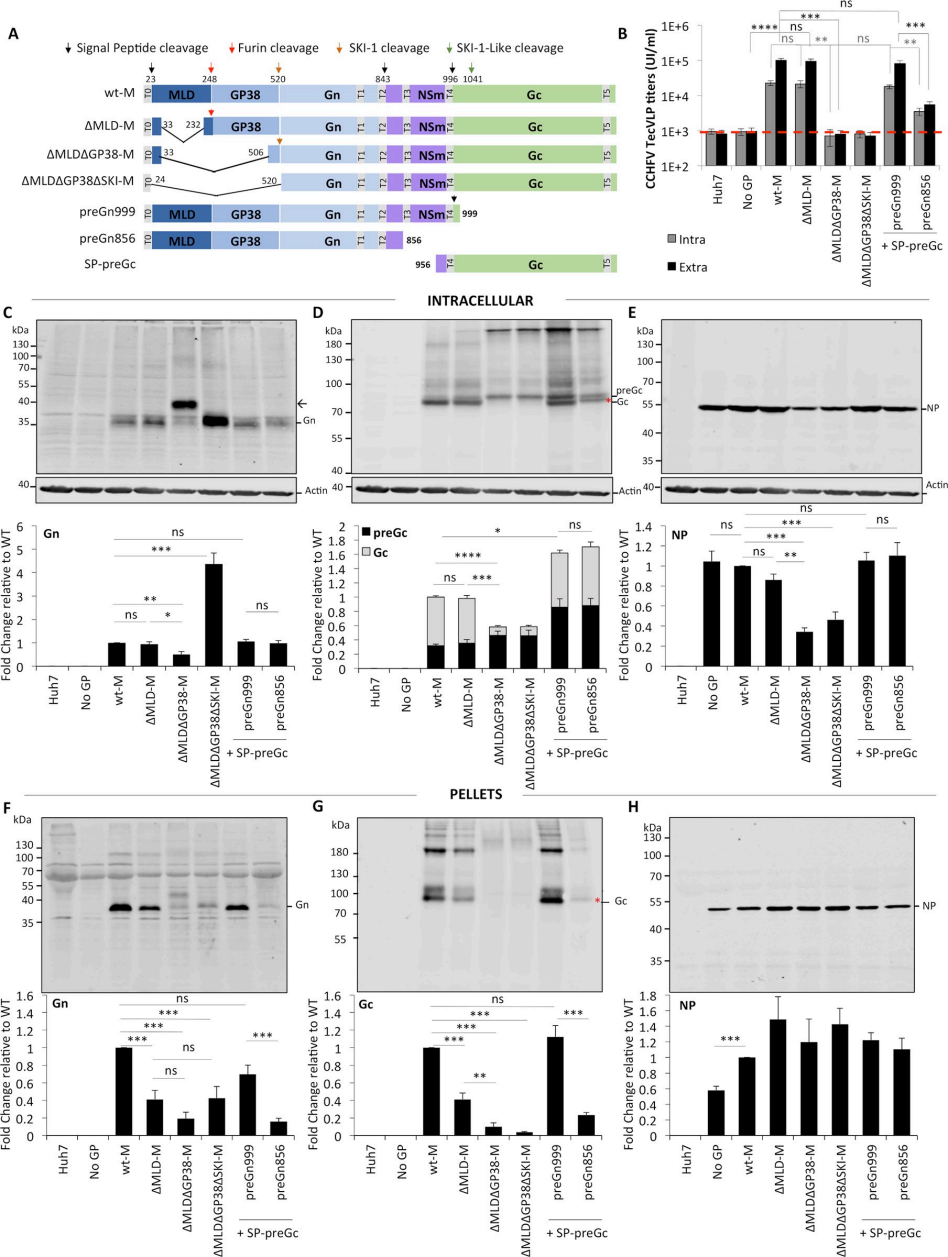
Effect of deleting the MLD, GP38 and NSm proteins from CCHFV M-segment on production of infectious CCHFV tc-VLPs. (**A**) Schematic representation of the polyproteins encoded by CCHFV wt-M cDNA and mutant cDNA clones: three internal deletion mutants (ΔMLD-M, ΔMLDΔGP38-M and ΔMLDΔGP38ΔSKI-M), two C-terminal truncation mutants (preGn999 and preGn856) and one N-terminal deletion mutant (SP-preGc). The polyprotein precursor organization and positions of the first amino-acid residues after cleavage marking protein boundaries (MLD, GP38, Gn, NSm, and Gc) within the CCHFV polyprotein are shown. The N-terminal signal peptide (T0) and putative transmembrane domains (T1 to T5) are shown as grey boxes, signal peptidase cleavage sites are indicated by black arrows and other host protein convertase cleavage sites are indicated by red and orange arrows. (**B**) Infectivity titers of intracellular and extracellular CCHFV tc-VLPs. At 72h post-transfection, clarified supernatants and cell-associated tc-VLPs were used to infect Huh7 cells pre-transfected with L and N, and titers were determined by FACS analysis 24h post-infection. (**C-E**) Intracellular levels of CCHFV proteins expression and processing. Cell lysates of tc-VLP-producing cells were analyzed by Western blotting with antibodies against the indicated proteins including Gn, Gc, NP and host actin. Intracellular protein band intensities were quantified and normalized relative to actin and expressed as fold change compared to wt-M. Representative western blot analysis and relative quantification of intracellular Gn (**C**), preGc and Gc (**D**), and NP (**E**) protein levels. (**F-H**) tc-VLP containing supernatants were concentrated by ultracentrifugation through 20% sucrose cushions, resuspended in Opti-MEM medium and analyzed by western blot. Representative blot analysis and relative quantification of Gn (**F**), Gc (**G**), and NP (**H**) expressed as fold change compared to wt-M. Molecular weight markers are marked on the left (kDa). Arrow correspond to Gn precursor raised from ΔMLDΔGP38-M construct and asterisks depict the shift in Gc migration. Statistical significance was determined using non-parametric two-tailed Mann–Whitney test. Average number of repeats for intracellular CCHFV proteins: Gn = 12 (6≤Gn≤19), Gc = 14 (6≤Gc≤22), NP = 11 (7≤NP≤16). Average number of repeats for CCHFV proteins in pellets: Gn = 11 (6≤Gn≤18), Gc = 13 (7≤Gc≤20), NP = 10 (6≤NP≤16). Average number of repeats for extracellular infectivity (E) was 10 (5≤E≤16) and intracellular infectivity (I) was 6 (3≤I≤9).

CCHFV, like all *orthonairoviruses*, carries a tri-segmented negative single stranded RNA genome in association with virus-encoded nucleoprotein (NP) and RNA-dependent RNA polymerase that are enclosed within a host-derived lipid bilayer studded with two viral envelope glycoproteins (GPs), Gn and Gc [3]. The genomic RNA segments are named according to their relative size, with the large (L) segment encoding the viral polymerase, the medium (M) segment encoding envelope-associated GPs (Gn and Gc) and several non-structural proteins, and the small (S) segment, through ambisense coding strategy, encoding the nucleoprotein (NP) and non-structural protein (NSs) [7]. CCHFV is the most genetically variable arthropod-borne virus. This diversity is highest in the single open reading frame (ORF) of the virus M segments exhibiting up to 31% and 27% of sequence divergence in nucleotide and amino-acid sequence, respectively [8]. CCHFV M segment encodes a single polyprotein precursor (Fig 1A), from which the two surface GPs are produced, but also several nonstructural proteins. The nascent polyprotein precursor features one leader N-terminal signal peptide (SP), two internal SPs and several trans-membrane domains (TMD) that are co-translationally processed by the endoplasmic reticulum (ER) signal peptidases (SPase). SP cleavage generates two intermediate precursors, preGn (140 kDa) and preGc (85 kDa) [9], and a double-membrane-spanning NSm protein of 15 kDa [10]. PreGn and preGc are subsequently post-translationally processed by host proteases to generate the mature virion envelope GPs, a mucin-like protein (MLD) containing a large number of predicted O-glycosylation sites and three secreted proteins of unknown functions: GP38, GP85, and GP160. The GP85/GP160 proteins contain both the GP38 protein and the MLD-like protein.

PreGc maturation to Gc (75 kDa) involves cleavage at the N-terminal RKPL^1040^ motif by an uncharacterized cognate SKI-1/S1P protease [11]. Gc is predominantly localized in the ER and requires direct or indirect interaction with Gn to enable its transport to the Golgi, the compartment of CCHFV assembly and budding [7]. PreGn contains a highly conserved Furin cleavage site motif RSKR^247^ located between the MLD and GP38, which appears unique to CCHFV, and a protease SKI-1/S1P cleavage site motif RRLL^519^ at the GP38 and Gn protein interface. SKI-1/S1P mediated cleavage, which releases Gn (37 kDa) from the C-terminus of GP38, occurs early in the secretory pathway and SKI-1/S1P is essential for CCHFV infectivity and incorporation of the viral glycoproteins in the virions [12]. N-glycosylation of MLD-GP38 initiated in the ER and further O-glycosylation in the Golgi are likely responsible for the occurrence of the protein in its two forms of different sizes (GP85 and GP160) [13]. In the late secretory pathway, more specifically in the trans-Golgi network (TGN), the RSKR^247^ motif is recognized and partially cleaved by Furin or Furin-like proprotein convertases (PC) separating the MLD protein from GP38 [13]. GP38, GP85 and GP160 are secreted from CCHFV infected cells as soluble proteins [13], and were recently shown to be targeted to the plasma membrane and to the virus envelope [14]. The functions of these proteins remain unknown but reduction of GP38 expression and secretion was shown to be associated with decreased preGn to Gn conversion and reduction of growth kinetics early during the infection [15].

The function of CCHFV NSm protein is currently unknown and is only predicted to exist in three other *orthonairovirus* species that exhibit close phylogenetic relationships to CCHFV (Dugbe, Hazara (HAZV), and NSDV) [4]. Interestingly, HAZV, which is nonpathogenic in humans and both phylogenetically and serologically related to CCHFV, uniquely features a 43 amino acid deletion in the cytoplasmic domain of NSm [4]. For orthobunyavirus members of the Peribunyaviridae family (order Bunyavirales), the NSm proteins have identical membrane topologies to that of CCHFV NSm, although no apparent sequence similarity exist. The NSm factor of the orthobunyavirus Bunyamwera virus (BUNV) was found to localize to the Golgi complex in infected cells and is involved in the formation of tubular virus-factories, which are essential for virus assembly and morphogenesis [16].

The pathogenesis associated with CCHFV infection is poorly understood but is likely derived from a complex interaction between the virus and host cells. Virus virulence is influenced by viral genes affecting viral spread and tropism. For instance, it was shown that GPs of different CCHFV strains have different ability to enter primary human cells [17]. Furthermore, CCHFV M segment-derived polyprotein processing is highly orchestrated and dependent on host factors restricted to specific organelles of the host secretory pathway. The nonstructural proteins traffic to the Golgi and co-localize with the envelope GPs at the sites of assembly and/or are secreted alongside with CCHFV virions from the infected cells, hence suggesting putative functions in CCHFV assembly and/or egress.

In this study, using a specific CCHFV minigenome-reporter transcription and entry-competent virus-like particle (tc-VLP) [18] production assay that mimics wild-type CCHFV [17, 18], we focused on the nonstructural proteins of the CCHFV M segment and their contributions to CCHFV assembly, secretion and infectivity. A thorough biochemical, imaging and functional analysis of tc-VLPs generated with a series of M segment deletion mutants allowed us to uncover important roles played by each nonstructural protein.

## Results

### NSm protein enhances CCHFV tc-VLP production while GP38 protein is an essential viral factor for formation of infectious particles

To evaluate the role of MLD, GP38 and NSm proteins, tc-VLPs were produced by co-transfection of Huh7 cells with a CCHFV minigenome-plasmid, CCHFV RNA polymerase (L) and nucleoprotein (NP) constructs to transcribe and replicate the minigenome, and a construct encoding proteins from either wild type M segment (wt-M) or one of the M deletion variants (Fig 1A). We generated three internally deleted mutants, in which the MLD protein alone (ΔMLD-M) or both the MLD and GP38 (ΔMLDΔGP38-M and ΔMLDΔGP38ΔSKI-M) were removed, and three truncation mutants: preGn999 and preGn856, which excluded Gc and both NSm and Gc, respectively, and SP-preGc, which only encoded the preGc precursor and was co-expressed with either former constructs. Expression of viral GPs in addition to the replicon construct leads to self-assembly into tc-VLPs, which structurally and functionally resemble the parental virus [18]. The produced particles recapitulate all steps of CCHFV replication cycle including virus entry that can be easily quantified by measuring the primary transcription of the reporter-GFP-minigenome post-infection by flow cytometry (FACS).

We first examined the ability of the generated mutants to support formation and release of intra- and extracellular infectious tc-VLPs by *in vitro* infection of target cells with cell-associated particles and crude supernatants of tc-VLP producer cells collected three days post-transfection (Fig 1B). As controls, non-transfected cells or cells transfected without GPs did not generate infectious particles, whereas expression of wt-M induced production of infectious tc-VLPs with average intra and extracellular titers of 2x10^4^ and 1x10^5^ IU/ml, respectively. ΔMLD-M allowed formation and release of infectious tc-VLPs at levels identical to wt-M. Contrastingly, neither intracellular nor extracellular tc-VLPs produced with ΔMLDΔGP38-M or ΔMLDΔGP38ΔSKI-M were infectious, suggesting that while CCHFV could tolerate deletion of the MLD protein, deletion the GP38 protein abolished production of infectious tc-VLPs. Furthermore, co-expression of preGn999 with SP-preGc produced infectious particles titers equaling those of tc-VLPs produced with wt-M, indicating that separation of CCHFV M segment into two units does not affect assembly, secretion and infectivity of tc-VLPs. Yet, NSm-deficient tc-VLP-producer cells co-expressing preGn856 and SP-preGc constructs produced lower amounts of intracellular infectious tc-VLPs (*ca*. 7-fold), suggesting defects in assembly or particle morphogenesis (Fig 1B). Finally, deletion of NSm resulted in 17 to 21 fold-reduction in the extracellular infectious tc-VLP titer when compared to preGn999/SP-preGc and wt-M derived tc-VLPs (4.9x10^3^ and 8.4x10^4^-1x10^5^ IU/ml, respectively), and strongly altered the ratio between intra- and extracellular infectious titers, suggesting retention of infectious particles.

Collectively, these results indicated that with exception of the MLD protein, both the GP38 and NSm proteins play important roles in formation and release of infectious tc-VLPs.

To get better insight into the functions of CCHFV M segment-encoded accessory proteins, we next analyzed the effect of deletion of MLD, GP38 and NSm non-structural proteins on Gn and Gc expression and processing, NP expression and secretion of particle-associated viral proteins. Whole-cell lysates of tc-VLP producer cells (defined as Intracellular in the figures) and the corresponding supernatants purified by ultracentrifugation through sucrose cushion (defined as Pellets in the figures) were analyzed by SDS-PAGE and Western blot at 3 days post-transfection (Fig 1C–1H). In agreement with previous studies [11, 18], expression of wt-M raised a mature Gn band at 37kDa (Fig 1C). Gc is expressed as a preGc precursor of 85kDa that is converted to mature Gc (75kDa) upon cleavage by a SKI-1 like protease [11]. The relative intracellular preGc and mature Gc levels are shown in Fig 1D. Expression of wt-M mostly raised mature Gc and a detectable 85kDa preGc precursor (*i*.*e*., less than 35%). Western blot analyses of the pellets of tc-VLP producer cell supernatants indicated that Gn and Gc were readily incorporated into particles with Gn being detected mostly as monomers, and Gc mostly detected as monomers and other higher molecular weight bands (Fig 1F–1G). NP intracellular expression and accumulation was not different between tc-VLP producer cells without GPs or transfected with wt-M, though NP incorporation into particles was increased when co-expressed with wt-M and the other deletion mutants (Fig 1E and Fig 1H).

Deletion of the MLD protein did not impact intracellular expression or processing of Gn, Gc and NP proteins (Fig 1C–1E), which is compatible with ΔMLD-M supporting assembly of infectious tc-VLPs with intracellular titers equivalent to wt-M (Fig 1B), but induced a reduction of 60% of both Gn and Gc in the pellet fractions without affecting NP secretion (Fig 1F–1H). Interestingly, the reduction in GPs levels did not appear to translate into lower infectivity titers. Besides infectious tc-VLPs, sub-viral particles (SVP) and non-infectious NP-containing particles deficient in viral GPs were produced and secreted during CCHFV replication, as shown by the detection of Gn- and Gc-containing particles upon transfection of wt-M construct alone (S1B Fig, lane 4) and by the release of NP-containing particles in the absence of GPs (Fig 1H and S1B Fig, lane 2). Decreased SVP formation and/or release or alternatively reduced GP incorporation are both possible explanations for the atypical ΔMLD-M mutant phenotype.

When both MLD and GP38 proteins were deleted, mature Gn was significantly reduced in tc-VLP producer cells expressing ΔMLDΔGP38-M segment, in which Gn release is mediated by SKI-1/S1P (Fig 1C). Instead of mature Gn (37kDa), a band of slower mobility of about 40kDa was mainly detected that most likely corresponded to a non-cleaved preGn “precursor” in which 25 residues of MLD and GP38 were retained in the ΔMLDΔGP38-M construct (Fig 1A and Fig 1C). Contrastingly, ΔMLDΔGP38ΔSKI-M from which Gn is cleaved by SP rather than processed by SKI-1/S1P raised increased intracellular mature Gn levels (>5-fold). Independently of the Gn intracellular levels, both ΔMLDΔGP38-M and ΔMLDΔGP38ΔSKI-M raised significantly lower total Gc levels (40% lower than wt-M), predominantly yielding a band corresponding to 85kDa preGc precursor (Fig 1D). Lower intracellular accumulation levels and predominant detection of preGc (80%) indicated that the GP38 protein encoded by the CCHFV M segment plays a role in preGc proteolytic processing. NP intracellular levels were also significantly reduced below 40% of those detected with wt-M in tc-VLP producer cells co-expressing ΔMLDΔGP38-M or ΔMLDΔGP38ΔSKI-M segments (Fig 1E).

Interestingly, preGc, which was poorly processed as mature Gc in ΔMLDΔGP38-M and ΔMLDΔGP38ΔSKI-M producer cells, was also poorly detected in the corresponding pellets, suggesting that preGc cleavage is required for Gc incorporation into particles. Deletion of GP38 also severely decreased incorporation of Gn into particles (Fig 1F). The fact that both double deletion M-segment mutants displayed a similar phenotype characterized by impairment of preGc processing strongly suggested that GP38 is an essential viral factor in processing and maturation of CCHFV surface glycoproteins.

Gn expression from the split constructs (preGn999 or preGn856) that also encode MLD-GP38 with Gc (SP-preGc) was indistinguishable from wt-M (Fig 1C). Expression of preGc from a plasmid encoding only the preGc ORF was more efficient than expression from wt-M, though mature Gc accumulated to levels similar to that of wt-M (70% of wt levels) (Fig 1D). These results suggested that preGc to Gc conversion is limited by Gn and GP38 levels rather than resulting from deficiencies in preGc processing. While deletion of NSm (preGn856 construct) did not affect cleavage and accumulation of CCHFV glycoproteins, it induced changes in the electrophoretic mobility of Gc (depicted by a red asterisk in Fig 1D and Fig 1G), implying that NSm can influence maturation of CCHFV GPs. In addition to the changes in protein electrophoretic mobility, deleting NSm protein induced more than 80% decrease of both Gn and Gc in tc-VLPs-containing pellets, which is consistent with the strong drop in the infectious titer (Fig 1B). Accordingly, differences in maturation of N-glycosylation can impact protein electrophoretic mobility and could be the reason for the differences observed. This hypothesis is addressed below and in Fig 6.

### GP38 is required for preGc processing and intracellular trafficking

**Fig 2:**
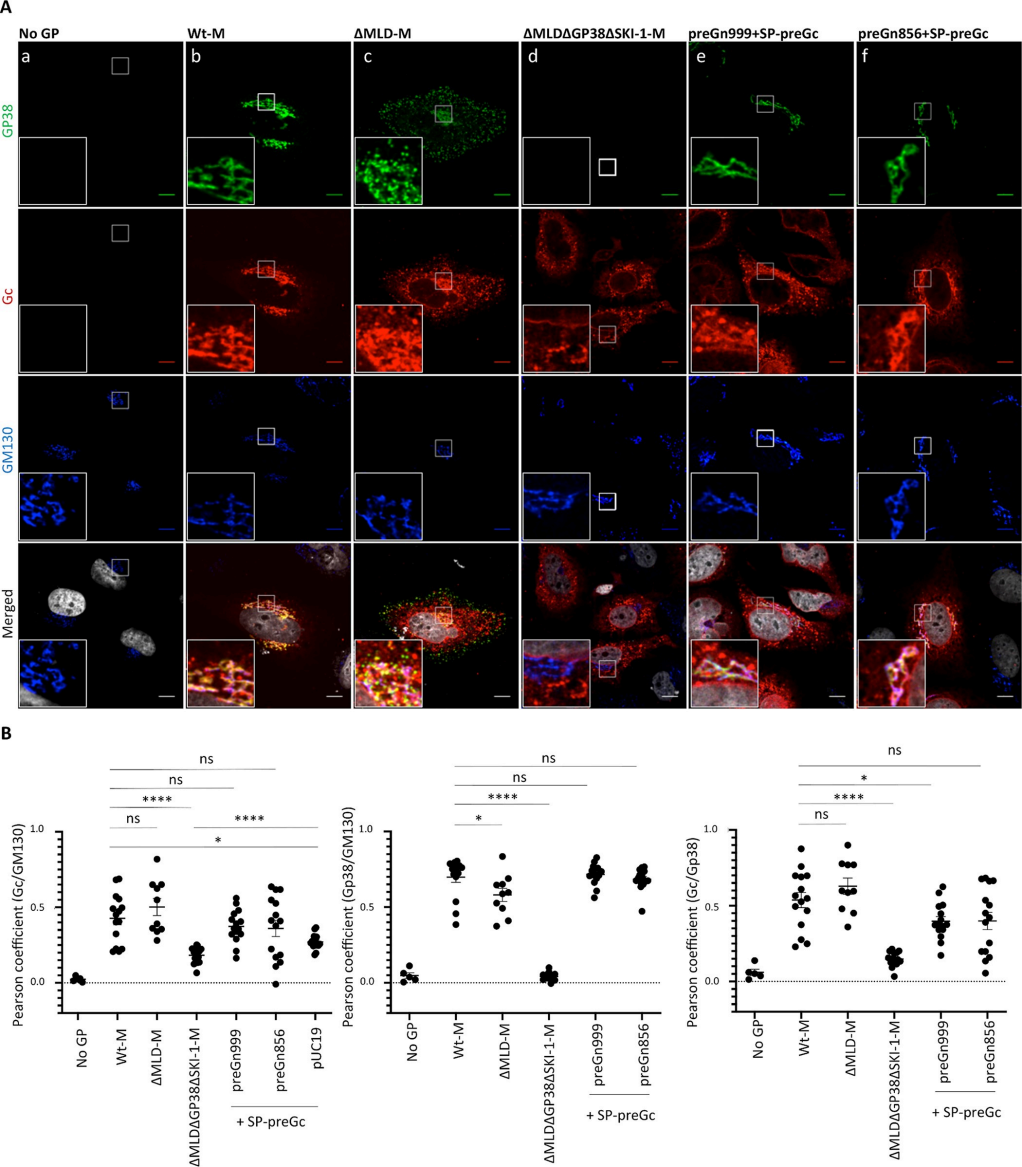
Golgi localization of CCHFV glycoprotein Gc is dependent on MLDGP38. (**A**) Confocal microscopy analysis of Huh7 cells transfected with pUC19-empty vector, wt-M, ΔMLD-M, ΔMLDΔGP38-M, a 1:1 mix of preGn999 or preGn856 (ΔNSm) with SP-preGc. At 48h post-transfection, cells were fixed, permeabilized with Triton X-100, and stained for GP38 (6B12, green channel), Gc (11E7, red channel), Golgi (anti-GM130, blue channel) and nuclei (Hoechst, grey channel). Magnification of the squared area is shown at the bottom of each condition, zooms from squared area represent 10μm. (**B**) Pearson’s coefficients were calculated using FIJI (JACoP) on 5 cells from 3 separated experiments (15 cells in total) and expressed as means ± SEM. Statistical significance was determined using non-parametric two-tailed Mann–Whitney test. Scale bars represent 10 μm.

The impact of deleting MLD, GP38 and NSm on intracellular localization of Gc was further studied by co-immunostaining with antibodies recognizing the Golgi organelle, the site of CCHFV assembly. Mature Gn intracellular distribution could not be analyzed since all previously isolated antibodies against Gn, such as 6B12 and 8F10 [19], are actually anti-GP38 antibodies (S2 Fig) [14]. As previously reported [19], Gc expressed from the wt-M construct partially colocalized with the GM130 cis-Golgi matrix protein (Fig 2A and Fig 2B), indicating that Gc is located inside or in close vicinity to the Golgi apparatus. In addition, it also exhibited a pattern compatible with ER localization (Fig 2A). Overall, Gc showed variation in the intracellular distribution pattern among transfected cells, as evidenced by the Pearson correlation coefficients (PCC) presented in Fig 2B. We also observed over time the formation of punctiform structures located in the cytoplasm (S3A Fig), in agreement with Gc synthesis in the ER and further trafficking through the Golgi. Deletion of MLD protein did not affect Gc intracellular localization whereas additional deletion of GP38 protein (both ΔMLDΔGP38-M constructs) resulted in a drastic reduction of Gc signal found in the Golgi, with PCCs similar to that of SP-preGc expressed alone shown in Fig 2. These imaging and biochemical analyses of Gc indicated that deletion of GP38 impedes the cleavage and transit of Gc from the ER to Golgi. Furthermore, these results showed that CCHFV Gn alone appears to be not sufficient to escort Gc from the ER to the Golgi and that GP38 is required to allow Gc cleavage and trafficking, hence likely explaining the failure to produce infectious tc-VLPs.

MLD-GP38/GP38 proteins produced from wt-M- or preGn999/SP-preGc-expressing cells strongly colocalized with GM130 protein, indicating that most of MLD-GP38/GP38 proteins are found in the Golgi apparatus (Fig 2). Interestingly, deletion of the MLD protein reduced GP38 colocalization with GM130 (Fig 2), which correlated with increased GP38 signal at the cell periphery, suggesting plasma membrane localization (Fig 2A). This phenotype was typically observed at later stages of wt-M expression (S3B Fig), suggesting an increase of secretion or plasma membrane retention of GP38 when MLD is absent. The former hypothesis is more probable since increased levels of GP38 were detected in the supernatants of tc-VLP-producer cells co-expressing GP38 when compared to those co-transfected with MLD-GP38 (S4D Fig)

Expression from the split constructs (preGn999 or preGn856 with SP-preGc) did not affect Gc localization, which was observed in the Golgi-like localization pattern of Gc derived from wt-M. Therefore, the reduced particle release associated with deletion of NSm cannot be attributed to an improper localization of Gc but rather to possible defects in the particle budding process and cell egress.

Altogether, through generation and analysis of CCHFV M segment accessory proteins deletion mutants, we identified GP38 and NSm as important viral factors for formation and release of infectious particles. GP38 deletion caused impairment of preGc cleavage and affected Golgi localization, ultimately preventing the formation of infectious particles. NSm deletion did not prevent formation of infectious particles or led to Gc mislocalization but rather induced retention of tc-VLP inside of the cell, resulting in reduced number of infectious particles released in the supernatant.

### Two different functions of MLD-GP38 assisting Gc processing and particle incorporation at different compartments of the secretory pathway

**Fig 3:**
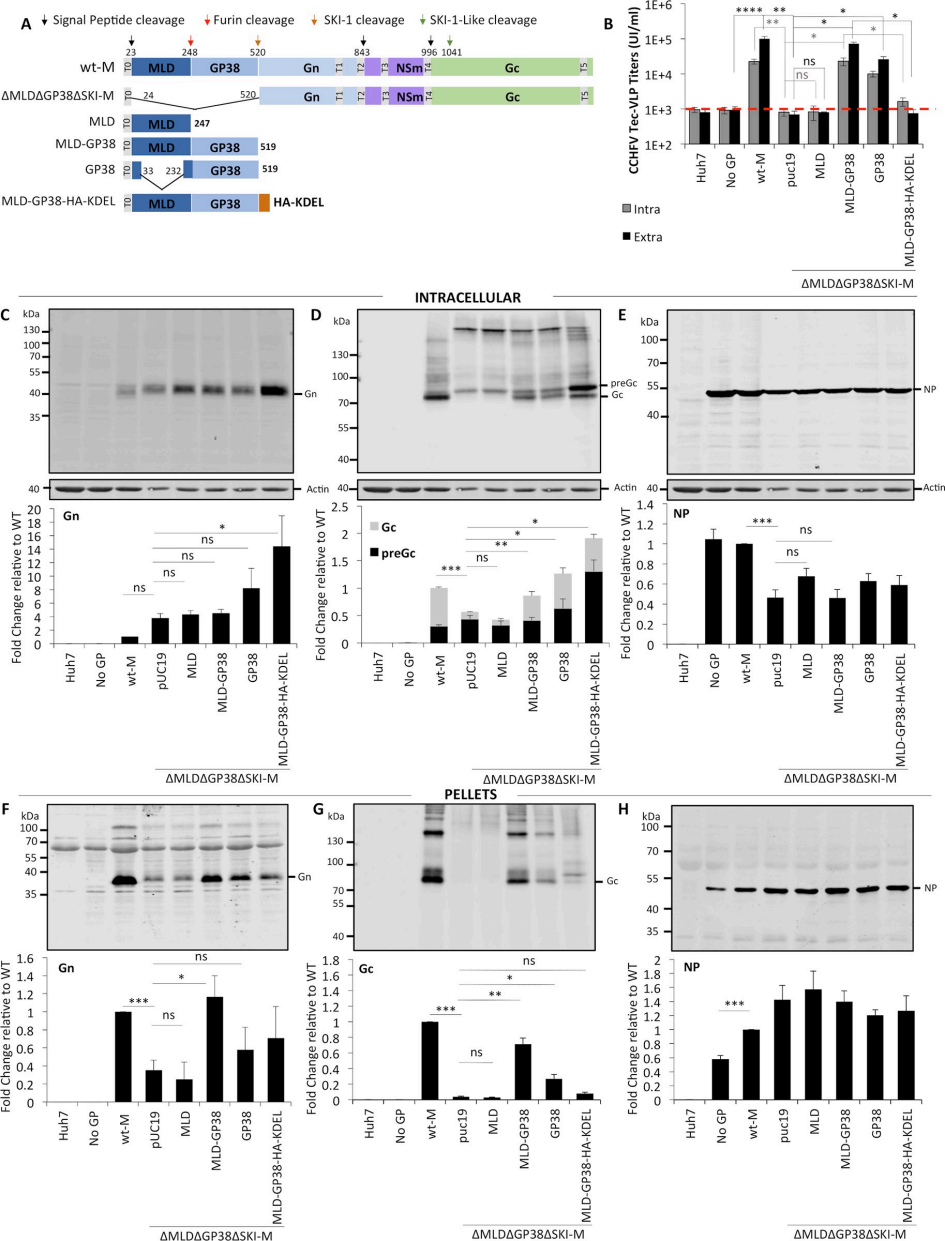
Rescue of defective tc-VLP infectivity by trans-complementation with wt-MLDGP38 or GP38. (**A**) Schematic representation of wt-M, ΔMLDΔGP38ΔSKI-M segment, MLD, MLD-GP38, MLD-GP38-KDEL and GP38 expressing constructs. CCHFV tc-VLPs were generated using constructs encoding either wt-M polyprotein, or ΔMLDΔGP38ΔSKI-M deletion mutant complemented in *trans* with either pUC19, or with MLD, MLD-GP38, MLD-GP38-HA-KDEL and GP38 expression vectors. Infectivity, CCHFV protein expression and tc-VLP secretion were analyzed at 72h post-transfection. (**B**) Clarified supernatants were inoculated on L and N pre-transfected Huh7 cells and infectious titers were determined by FACS 24h post-infection. (**C-E**) Intracellular levels of CCHFV proteins expression and processing. Cell lysates of tc-VLP-producing cells were analyzed by Western blotting as described in Fig 1. Representative western blot analysis and quantification of intracellular Gn (**C**), Gc (**D**) and NP (**E**) protein levels. (**F-H**) tc-VLP secretion. Western blot analysis of tc-VLP-associated proteins purified by ultracentrifugation through 20% sucrose cushion. Representative blot analysis and relative quantification of Gn (**F**), Gc (**G**) and NP (**H**) expressed as fold change relative to wt-M. Molecular weight markers are marked on the left. Statistical significance was determined using non-parametric two-tailed Mann–Whitney test. Average number of repeats for intracellular CCHFV proteins: Gn = 8 (3≤Gn≤19), Gc = 9 (3≤Gc≤22), NP = 8 (3≤NP≤16). Average number of repeats for CCHFV proteins in pellets: Gn = 8 (4≤Gn≤18), Gc = 9 (3≤Gc≤20), NP = 8 (4≤NP≤16). Average number of repeats for extracellular infectivity (E) was 8 (3≤E≤16) and intracellular infectivity (I) was 5 (3≤I≤9).

To further understand the functions of GP38, we next designed a trans-complementation assay of a MLD-GP38-deleted M segment with GP38 and/or MLD expression constructs (Fig 3A). This allowed the analysis of whether MLD-GP38/GP38 proteins outside of the M segment-encoded polyprotein and thus, independently of processing, could restore GPs processing and Golgi localization, and rescue production of infectious particles. We found that either MLD-GP38 or GP38 alone dose-dependently restored expression and preGc/Gc ratios (Fig 3D and S4A Fig), improved Gc incorporation into purified viral particles (Fig 3G and S4C Fig) and infectivity to near-wt levels (Fig 3B and S4B Fig). In contrast, complementation with MLD had no effect on Gn or preGc synthesis and/or accumulation and did not rescue Gc proteolytic processing defect (Fig 3C and Fig 3D), GPs incorporation (Fig 3F and Fig 3G) and infectivity (Fig 3B). These results suggested that MLD-GP38/GP38 act as molecular chaperones assisting folding and/or regulating hetero-oligomerization between Gn and Gc, which could be important for processing and trafficking of Gc.

MLD-GP38/GP38 kinetics of expression and secretion, and intracellular localization (Fig 2A) closely mirrors that of Gn and Gc [9, 13]. To address if MLD-GP38 targeting to the Golgi and secretion is important for tc-VLP assembly and release, we tested in the above-described tc-VLPs trans-complementation assay an ER-retained variant of MLD-GP38 fused at the C-terminus with a KDEL motif (MLD-GP38-HA-KDEL), which prevents cargo secretion from ER/Golgi by retrograde transport [20]. MLD-GP38-HA-KDEL was not detected in the cultured media indicating ER retention (S5 Fig). Trans-complementation with MLD-GP38-HA-KDEL raised the expression levels of both Gn and Gc, and partially restored preGc to Gc conversion (Fig 3C and Fig 3D). Yet, while virion incorporation of Gn was as high as in trans-complementation with GP38-only (Fig 3F), both Gc and preGc were poorly detected in tc-VLPs (Fig 3G), and infectivity could not be restored (Fig 3B). Although, some MLD-GP38-HA-KDEL leakage from the ER *via* saturation of KDEL-receptors cannot be excluded, these results indicated that trafficking of MLD-GP38 beyond the ER, intermediate compartment and efficient targeting to the Golgi, while dispensable for Gc processing, is important for incorporation of correctly sized mature Gc.

Generation of recombinant CCHFV particles in which the RSKR^247^ Furin cleavage site located between MLD and GP38 is mutated showed that cleavage of the M-polyprotein at this RSKR motif is dispensable for CCHFV propagation [15]. Mutagenesis of arginine residues at positions P1 and P4 of this Furin cleavage site blocked glycoprotein processing to GP38 and slightly reduced PreGn to Gn conversion, suggesting that either GP38 and/or reduced Gn maturation accounted for the reduction of CCHFV titers, by about 10-fold [15], albeit the hypothesis was not further explored. To clarify if MLD-GP38 processing to GP38 is important for CCHFV production, we generated two M segment constructs encoding CCHFV glycoprotein precursors lacking a functional Furin cleavage motif (RSKR mutated to either ASAA or QSQQ: M-ASAA and M-ASQQ constructs in S6A Fig). Also, identical mutations were introduced in the MLD-GP38 expression constructs (MLD-GP38-ASAA and MLD-GP38-QSQQ mutants) and analyzed in trans-complementation with the ΔMLDΔGP38ΔSKI-M construct. The trans-complementation assay should allow discriminating the effects of blocking GP38 maturation from preGn processing, since Gn expression from ΔMLDΔGP38ΔSKI-M construct is increased by up to 5-fold (Fig 1C and Fig 3C).

Mutating RSKR to ASAA or QSQQ impaired maturation of the glycoprotein precursor to GP38 (S6I Fig and S6J Fig) in a stronger manner than a RSKR to ASKA mutation where trace amounts of GP38 were still detected in the supernatants [15]. Blocking glycoprotein precursor’s maturation to GP38 resulted in a 5- to 6.4-fold reduction of CCHFV tc-VLP titers (S6B Fig), which correlated with reduction of GPs detected in the pellet fractions (S6F Fig and S6G Fig). This reduction was less abrupt for the tc-VLPs produced by trans-complementation of ΔMLDΔGP38ΔSKI-M with MLD-GP38ASAA and MLD-GP38QSQQ mutants (2.7- to 3.1-fold reduction, when compared to tc-VLPs produced by trans-complementation with wt-MLD-GP38, S6B Fig). Overall, no significant differences were detected in the intracellular Gn and Gc protein expression levels that could explain the reduction in titers (S6C Fig and S6D Fig) Blocking GP38 processing had no negative effect on preGc to Gc conversion either in the context of the full M segment polyprotein (*i*.*e*., M-ASAA and M–QSQQ) or in the trans-complementation assay, since both MLD-GP38-ASAA and MLD-GP38-QSQQ mutants supported preGc cleavage and Gc maturation as efficiently as the wt MLD-GP38 (S6C Fig). While we cannot exclude a delay in PreGn to Gn conversion due to mutating Furin cleavage site, it appears that GP38 maturation enhances tc-VLP assembly and/or GPs incorporation, though it not essential for formation of infectious tc-VLPs.

### Gc localization in the Golgi is important for efficient formation of infectious tc-VLPs and is regulated by MLD-GP38

**Fig 4:**
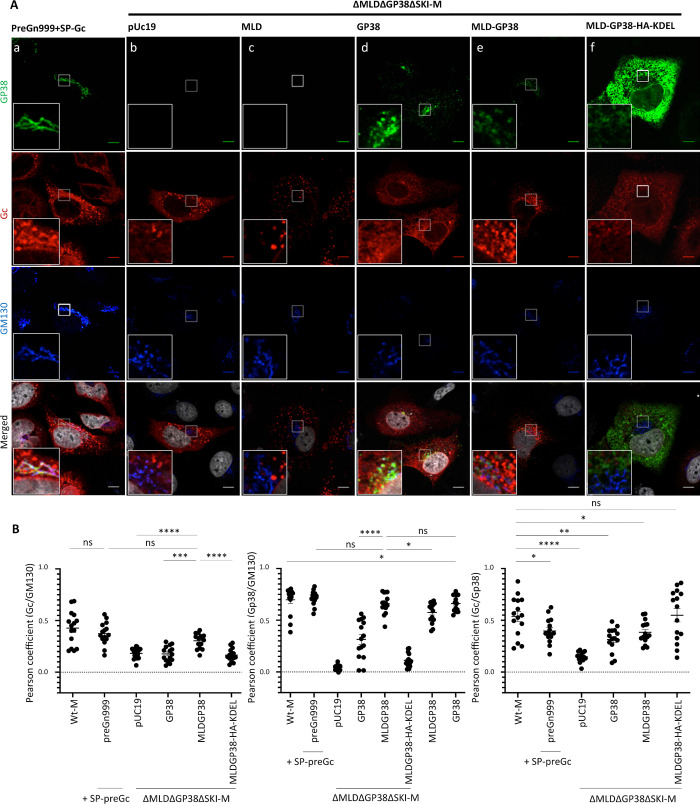
GP38 and Gc co-localization in the Golgi is required for rescue of tc-VLP production with ΔMLDΔGP38ΔSKI-M segment. (**A)** Confocal microscopy analysis of Huh7 cells transfected with different expression plasmids as indicated. At 48h post-transfection, cells were fixed, permeabilized with Triton X-100, and stained for GP38 (6B12, green channel), Gc (11E7, red channel), Golgi (anti-GM130, blue channel) and nuclei (Hoechst, grey channel). Magnification of the squared areas are shown at the left bottom of each condition. (**B**) Pearson’s coefficients were calculated using FIJI (JACoP) on 5 cells from 3 separated experiments (15 cells in total) and expressed as mean (and SEM). Statistical significance was determined using non-parametric two-tailed Mann–Whitney test. Scale bars represent 10μm.

Next we analyzed if the rescue of tc-VLP infectivity by GP38-expressing constructs was correlated with Gc intracellular localization (Fig 4). MLD-GP38-HA-KDEL and Gc proteins showed similar intracellular distribution, though both proteins were excluded from the Golgi (Fig 4). The staining pattern of the ectopically expressed MLD-GP38 and GP38 closely resembled that of GP38 raised from wt-M, with both proteins being mainly detected in the Golgi (Fig 4B and S2 Fig). Co-expression with ΔMLDΔGP38ΔSKI-M did not dramatically affect the intra-cellular localization of MLD-GP38, albeit it seemed to increase MLD-GP38 targeting to the Golgi. Contrastingly, co-transfection of ΔMLDΔGP38ΔSKI-M and GP38 expression constructs altered the distribution of GP38, which was less associated with the Golgi and frequently detected at the cell periphery, mimicking the pattern observed in tc-VLP-producer cells transfected with ΔMLD-M deletion mutant (Fig 2 and Fig 4).

Both MLD-GP38/GP38 proteins expressed ectopically were localized close to Gc, though without any obvious co-localization. Despite this, Gc localization in the Golgi was significantly increased in MLD-GP38 co-expressing cells (Fig 4), irrespectively of both MLD-GP38 and GP38 proteins being efficient in supporting release of infectious tc-VLP (Fig 3). The overall low co-localization between Gc and GP38 in trans-complementation may suggest that the interactions between these two proteins are favored before PreGn maturation, and, upon cleavage by SKI-I, that MLD-GP38 dissociates from the protein complexes formed by Gn and Gc. Consistent with this hypothesis, neither deletion of the MLD protein nor mutating the RSKR^247^ Furin cleavage site between MLD and GP38 to QSQQ in the M segment caused changes in Gc and GP38 co-localization (Fig 2B and S7 Fig). Whereas Furin cleavage at RSKR^247^ did not or only slightly modified the intracellular distribution of GP38, it induced a slight decrease in Gc and GM130 co-localization (S7 Fig). Overall, blocking M polyprotein precursor processing to GP38 appeared to regulate Gc localization in the Golgi while the MLD and Gn/Gc proteins seem to control GP38 localization. Thus, these results suggested that co-localization of MLD-GP38 and Gc and trafficking to the Golgi is essential for production of infectious particles.

### CCHFV NSm protein affects Gc maturation and facilitates tc-VLP secretion

**Fig 5:**
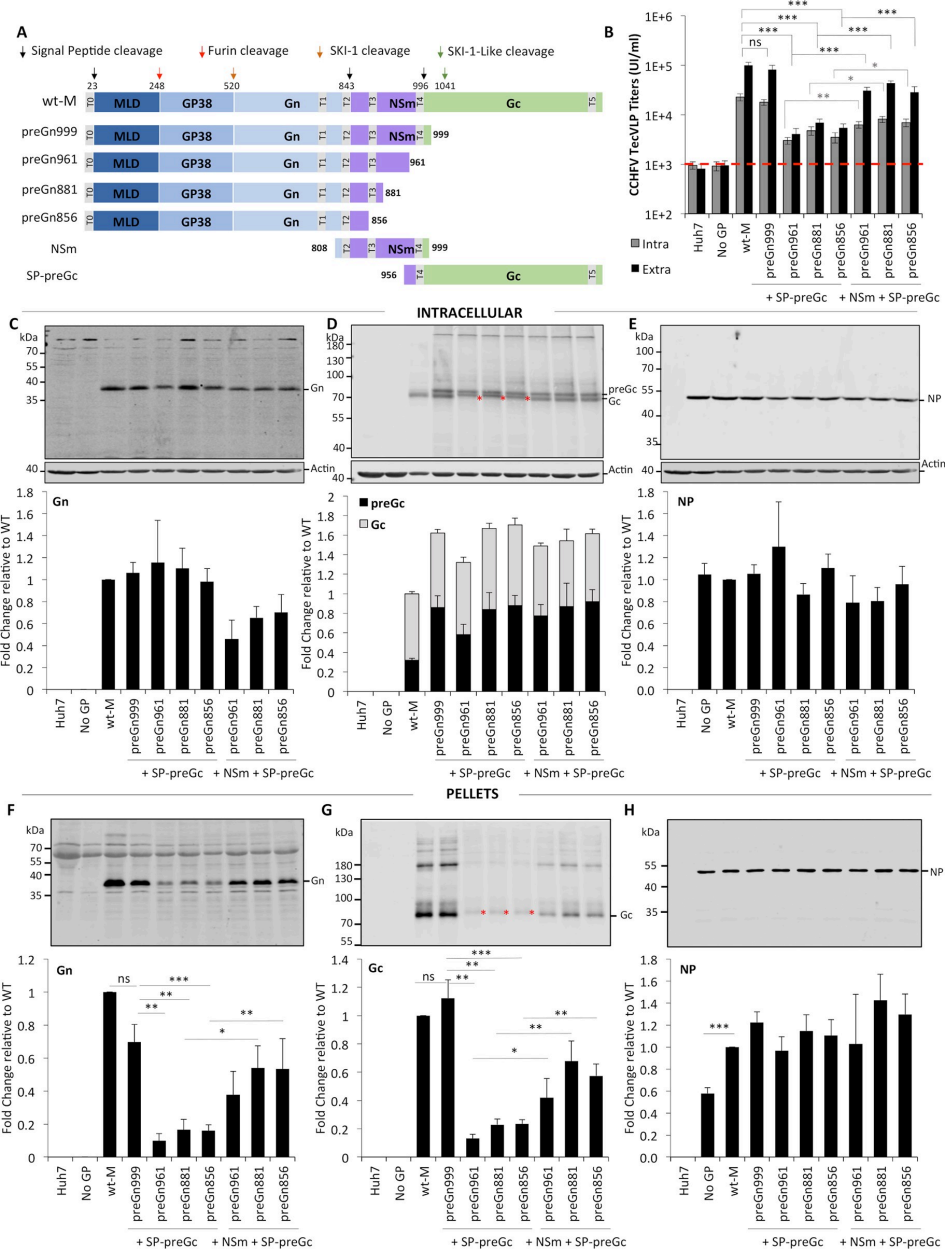
Expression of NSm in *trans* restores the assembly and secretion of tc-VLPs originated with preGn/Nsm C-terminal truncation mutants. (**A)** The polyprotein precursor layout encoded by the wt-M segment is shown at the top. Below are shown schematics of i) preGn999, ii) three preGn/NSm truncation mutants with progressively shorter NSm sequence ending at amino-acid position 961, 881 and 856, iv) NSm and SP-preGc expression constructs. Infectivity, CCHFV protein expression and tc-VLP secretion was analyzed at 72h post-transfection. (**B**) Infectious titers of intra and extracellular CCHFV tc-VLPs. Clarified supernatants and cell-associated tc-VLPs isolated after three freeze-thaw cycles were used to infect Huh7 cells pre-transfected with L and N, and titers were determined by FACS analysis 24h post-infection. (**C-E**) Intracellular levels of CCHFV proteins expression and processing. Cell lysates of tc-VLP-producing cells were analyzed by Western blotting as described in Fig 1. Representative western blot analysis and relative quantification of intracellular Gn (**C**), preGc and Gc (**D**), and NP (**E**) protein levels. (**F-H**) tc-VLP secretion. tc-VLP containing supernatants were first concentrated by ultracentrifugation through 20% sucrose cushions, and analyzed by western blot. Representative blot analysis and relative quantification of Gn (**F**), Gc (**G**) and NP (**H**) expressed as fold change compared to wt-M. Asterisks depict the shift in Gc migration. Statistical significance was determined using non-parametric two-tailed Mann–Whitney test. Average number of repeats for intracellular CCHFV proteins: Gn = 9 (3≤Gn≤19), Gc = 9 (3≤Gc≤22), NP = 8 (3≤NP≤16). Average number of repeats for CCHFV proteins in pellets: Gn = 9 (4≤Gn≤18), Gc = 10 (4≤Gc≤20), NP = 8 (3≤NP≤16). Average number of repeats for extracellular infectivity (E) was 10 (7≤E≤16) and intracellular infectivity (I) was 7 (4≤I≤9).

Having shown that the deletion of almost the entire NSm protein sequence (preGn856 construct) affected Gc maturation, slowing down its electrophoretic mobilities (Fig 1D), and reduced tc-VLP assembly, secretion and infectivity (Fig 1B and Fig 1F–1G), we next analyzed whether alternative constructs in which the first NSm TMD (preGn881 construct) or both first TMD and cytosolic domain (preGn961 construct) were introduced could restore the above (Fig 5A).

As depicted in Fig 5C, all NSm truncation mutants co-expressed with SP-preGc raised mature Gn at levels similar to those of wt (*i*.*e*., with wt-M or preGn999/SP-preGc constructs) with small expression differences of 2-fold or lower. Likewise, equivalent levels of mature Gc were detected (between 1.2- to 1.5-fold variation), whereas preGc was readily detected at levels augmented relative to wt-M (Fig 5D). Similarly, NP intracellular expression was uniform with little difference in NP accumulation levels, ranging from 0.8- to 1.3-fold as high as in wt (Fig 5E). Both Gn and Gc proteins from cells lacking NSm expression showed slower electrophoretic mobility (Fig 5C and 5D).

Truncation of the C-terminus of NSm, independently of the extent of the deletion, constantly resulted in up to 8-fold reduction of both Gn and Gc incorporation into particles relative to wt-M or to preGn999/SP-preGc levels, without affecting secretion of NP (Fig 5F–5H). Concomitant with the defects in secretion, tc-VLPs generated with the NSm truncated constructs were *ca*. 14-24-fold less infectious than wt (Fig 5B). Altogether, these results indicated a requirement of the full NSm protein for efficient assembly and secretion of infectious tc-VLPs. In support of this hypothesis, co-transfection of an NSm-expression construct (Fig 5A) with SP-preGc and either one of the NSm truncation mutants was sufficient to restore wt Gc migration pattern (Fig 5D) and to partially restore particles secretion and infectivity of tc-VLPs (Fig 5B and Fig 5F–5H).

### NSm accelerates protein trafficking through the secretory pathway with alterations in protein N-glycosylation

NSm-induced alteration of Gc electrophoretic mobility (Fig 5) could be due to modification of its glycosylation profile. However, Gc contains only two N-glycan sites at positions 1054N and 1563N [21] and the available antibody (mAb 11E7) for western blotting requires no denaturation and absence of non-reducing agents [19], which could prevent total deglycosylation using PNGase F that is sensitive to protein conformation. Therefore, we investigated whether NSm-dependent effects on Gc mobility are specific to CCHFV glycoproteins or could also influence the mobility of other glycoproteins, and if the changes in protein migration are due to altered N-glycosylation.

**Fig 6:**
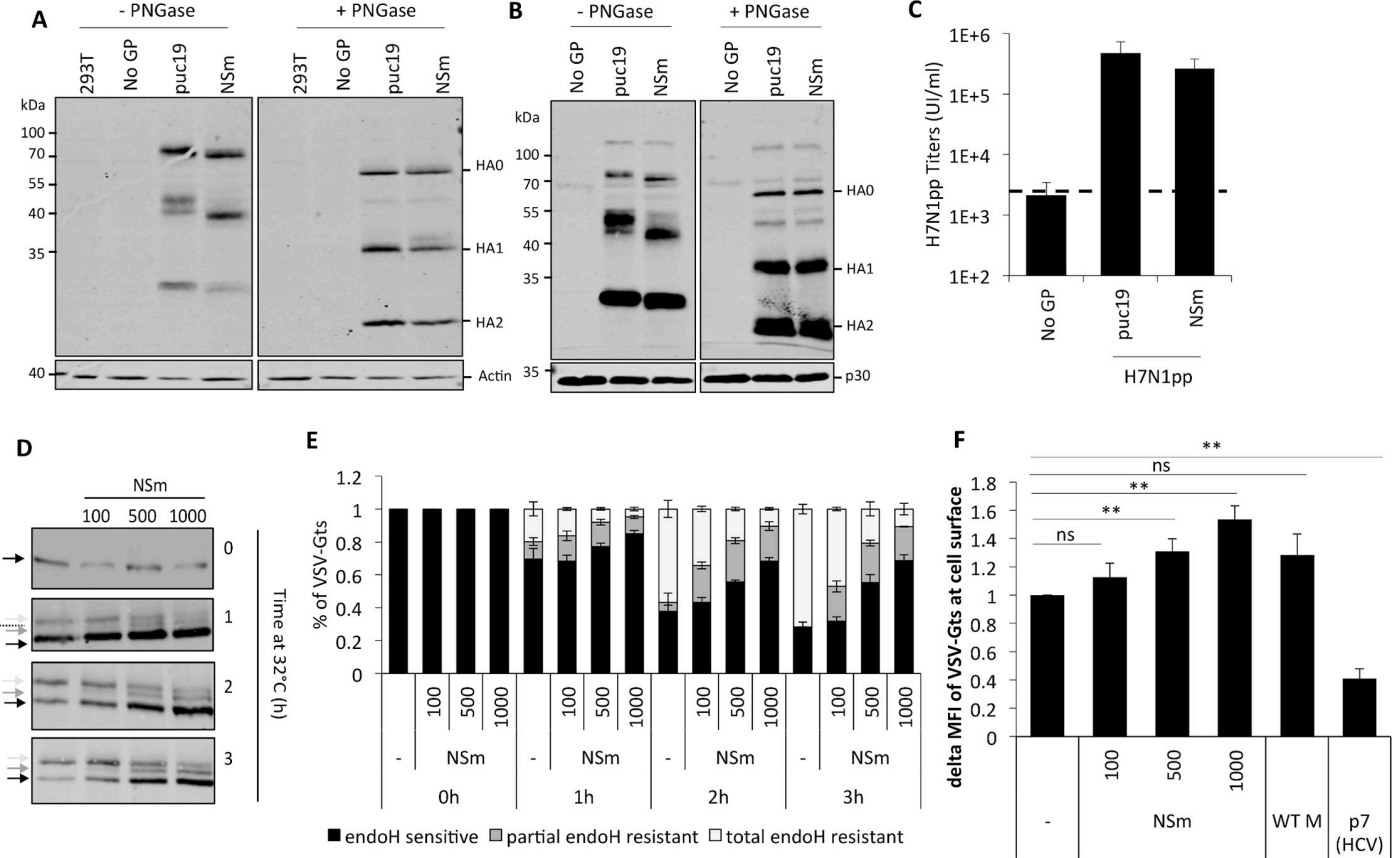
CCHFV NSm alters the glycosylation of viral glycoproteins and accelerates VSV-G trafficking in a dose dependent fashion. (**A-C)** Mobility shift of hemagglutinin (HA) of H7N1 induced by co-expression of NSm. Cell lysates and supernatants of H7N1pp-producer cells were analyzed by western blot after treatment with PNGase F (enzyme free and PNGase F-treated samples were migrated on the same SDS-PAGE gel), using antibodies against HA and host actin (**A-B**). Infectious titers of H7N1pp titers (**C**). (**D-F**) Impact of NSm on VSV-Gts endoH resistance and intracellular trafficking kinetics. Huh7 were transfected with plasmids encoding for VSV-Gts and different doses of NSm (100 ng, 500 ng and 1,000 ng) or HCV p7 when specified. Representative western blot and quantification analysis of cell lysates digested with endoH (**D-E**). Cell surface expression of VSV-G 1h at 32°C by flow cytometry, using the 41A1 mAb directed against VSV-G ectodomain (**F**). The values represent the variations of the mean fluorescence intensity (delta MFI) of cell surface-expressed VSV-Gts relative to time 0h at 32°C and relative to control condition. Data represent mean values ± SEM. Significance values were calculated by applying the unpaired one sample t-test using the GraphPad Prism 6 software (GraphPad Software, USA).

First, we examined if NSm altered H7N1 influenza virus HA glycoproteins expression and processing as well as production, secretion and infectivity of H7N1 pseudoparticles (H7N1pp), which are assembled at the plasma membrane [22]. Similar to CCHFV glycoproteins, H7N1 HA proteins are synthesized in the ER as a polyprotein that undergoes N-glycosylation, moves through the Golgi apparatus and is cleaved by Furin in the TGN. NSm expression in H7N1pp-producing cells had no major effect on expression levels of the HA precursor (HA0) and on its cleavage into HA1 and HA2 proteins as compared to control H7N1pp producer cells (Fig 6A and Fig 6B). Yet, while HA1 was detected as two species of different sizes, likely due to different N-linked glycans, only one HA1 species migrating with a faster mobility was detected in both lysates and pelleted H7N1pp from NSm co-expressing cells (Fig 6A and Fig 6B). Slighter changes in migration of HA0 and HA2 were also detected (Fig 6A and Fig 6B). Treatment with PNGase F, which removes N-linked oligosaccharides, of both lysates and supernatants of H7N1pp producer cells confirmed that the electrophoretic mobility of HA1 and the more subtle differences in migration of HA0 and HA2 were due to altered glycosylation induced by NSm expression (Fig 6A and Fig 6B). These results suggested a general NSm-dependent alteration of protein N-glycosylation. Interestingly, this alteration had no effect on H7N1pp production and secretion, as evidenced by equivalent secreted protein levels and infectious titers yielded by producer cells expressing NSm or pUC19-empty vector (Fig 6C), hence suggesting that NSm could alter the N-glycosylation pathway without affecting the trafficking of glycoproteins.

Since the lack of effect of NSm on infectivity and amounts of secreted H7N1pp could be due to steady-state detection conditions, we sought to investigate the effect of NSm on the rate of trafficking through the secretory pathway. Accordingly, we used the ts045 temperature-sensitive mutant of vesicular stomatitis virus G protein (VSV-Gts), which is misfolded and retained in the ER at restrictive temperature (40°C) [23]. VSV-Gts was co-expressed with increasing amounts of NSm and, after temperature shift from 40°C to 32°C (which allows refolding and release of VSV-Gts from the ER to the Golgi) cell lysates collected at different points were treated with endoglycosidase H (endoH), an enzyme that removes mannose rich (ER/cis-Golgi form) but not hybrid or complex (Golgi and post-Golgi form) N-linked oligosaccharides. While before temperature shift, all VSV-Gts were endoH-sensitive, reflecting ER retention at 40°C, VSV-Gts progressively became less sensitive to endoH digestion (Fig 6D and Fig 6E), reflecting, as expected, the maturation of the two glycans of each glycoprotein, concomitant to its refolding and transport across the Golgi network. Outstandingly, we found that NSm affected the kinetics and levels of VSV-Gts sensitivity to endoH digestion. Indeed, NSm co-expression resulted in a dose-dependent decrease of the kinetics of VSV-Gts endoH-resistance (Fig 6D and Fig 6E). In addition to increased levels of endo-H sensitive VSV-Gts, a third form of VSV-Gts harboring both a mature and immature glycan was detected in cells co-expressing NSm. These results could reflect the alteration of N-glycosylation maturation induced by NSm as observed for HA glycoproteins. Alternatively, they could also suggest ER retention or delay in the ER-to-Golgi and/or intra-Golgi trafficking, leading to a decrease in the rate of N-glycan processing and maturation.

Finally, to get more insight into these hypotheses and try to disentangle NSm effect on N-glycoslylation maturation from possible effects on transport, we monitored the ER-to-plasma membrane transport kinetics of VSV-Gts co-expressed with increasing amounts of NSm or control constructs. HCV-p7, used as an ER-retention factor, slowed down the rate of VSV-Gts trafficking, resulting in a decrease of the reporter protein at the cell surface, as previously reported [24]. In contrast, upon co-expression of NSm VSV-Gts cell surface expression was increased in a dose-dependent manner, suggesting that NSm expression accelerates protein trafficking (Fig 6F).

Altogether, these results indicated a general effect of NSm on protein N-glycosylation that is not linked to ER retention or to delay in trafficking through the secretory pathway. This dual effect suggested that NSm acts as an agonist of protein trafficking with antagonistic effect on N-glycosylation, which are both important for CCHFV assembly and secretion.

## Discussion

The biogenesis of enveloped viruses is tightly linked to the molecular processes regulating synthesis, trafficking and maturation of their surface glycoproteins. These proteins are key determinants regulating viral infectivity, as they mediate virus entry *via* interaction with attachment factor(s) and receptor(s), virus-host membrane fusion events, and subsequent dissemination by enabling assembly and release of progeny particles. Previous studies uncovered that CCHFV utilizes host SPases and hijacks proprotein convertases (PC) with different substrate specificity and/or spatial distribution to generate mature Gn and Gc proteins, the building blocks of its envelope, as well as five non-structural viral proteins [11–13]. Changes in GP160 and GP85 to GP38 protein expression ratios by mutagenesis of the Furin substrate motif (RSKR), located between MLD and GP38, resulted only in a transient reduction of infectious CCHFV titers, suggesting that either Furin cleavage or GP38 protein is dispensable for viral spread [15], albeit GP38 was still expressed in the form of GP160 and GP85 proteins. Besides these reports, relatively little is currently known on the functions of the non-structural proteins encoded by the CCHFV M segment, which calls for further studies to better understand how they intervene in assembly and virus transmission.

**Fig 7:**
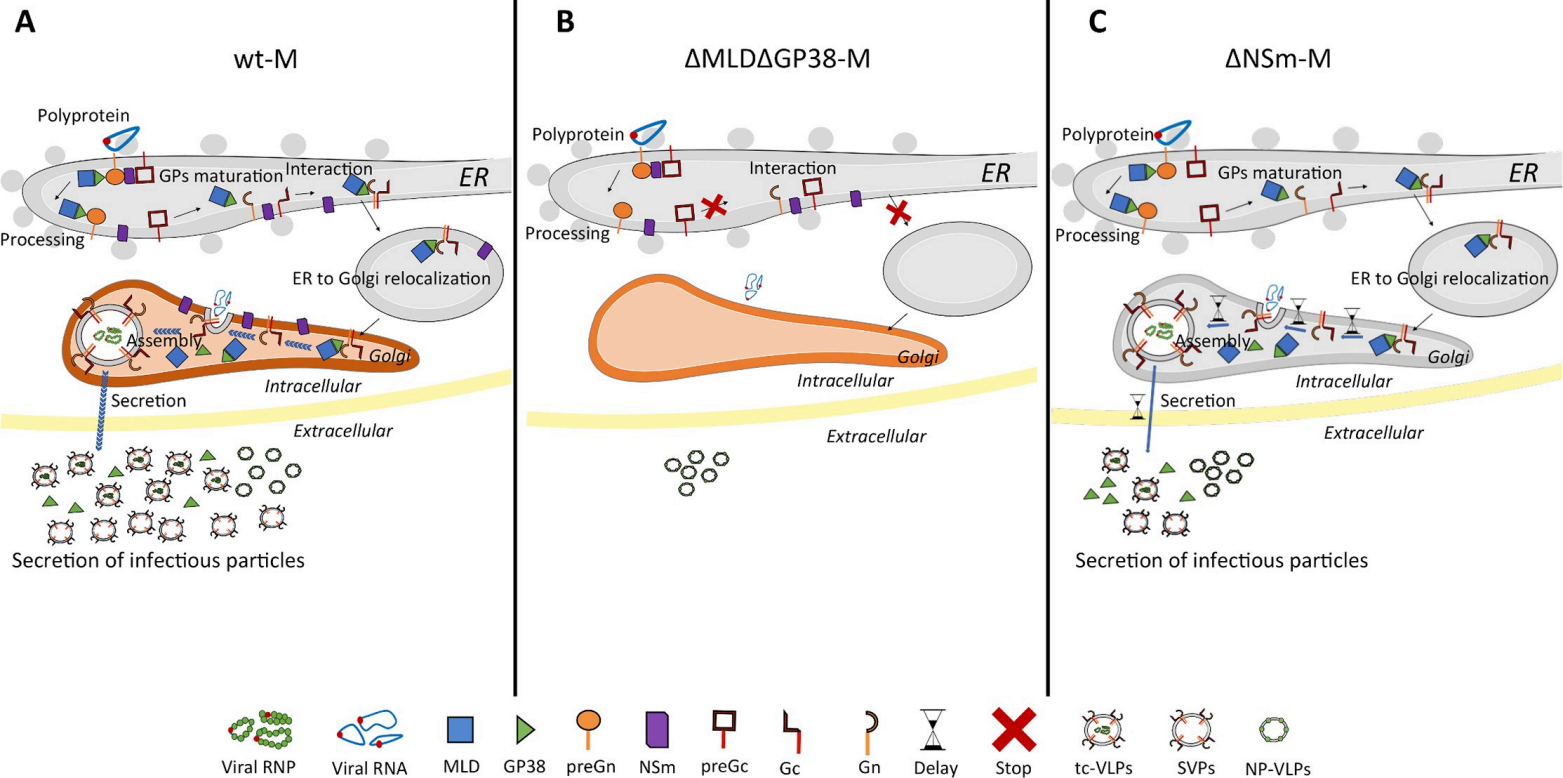
Working model of GP38 and NSm proteins functions in CCHFV glycoprotein processing, virion assembly and secretion. (**A**) CCHFV M segment encoded polyprotein is co-translationally cleaved at three sites by the ER SPase releasing Gn and Gc precursors (preGn and preGc) and the membrane associated NSm protein. PreGc to Gc conversion and Golgi targeting requires association with either preGn or mature Gn released by SKI-1/S1P in complex with GP85/GP38. Accumulation of GPs in the Golgi and interactions with the virus RNPs leads to assembly and budding of progeny virions into the Golgi cisternae and SVPs. (**B**) Deletion of GP38 likely prevents Gn and preGc association resulting in impairment of preGc processing, intracellular trafficking and consequently abrogation of virion and SVP assembly. (**C**) Efficient particle secretion involves manipulation of the secretory pathway by NSm, which increases the rate of protein trafficking and alteration in protein N-glycosylation profiles. Deletion of NSm results in defects in virion and SVP assembly and/or incomplete particle morphogenesis due to retention of progeny virions in the Golgi cisternae. Formation and secretion of NP-VLP as in B) and C) is unchanged because it is independent of CCHFV GPs biogenesis and trafficking.

In this report, through mutagenesis, biochemical, imaging and functional analysis, we investigated whether MLD, GP38 and NSm proteins of the CCHFV M segment are critical *vs*. dispensable for CCHFV infectivity. Using this combined approach, we demonstrated that all M segment-encoded accessory proteins play roles at least in one of the steps governed by the CCHFV envelope proteins, *i*.*e*. assembly, secretion and infectivity, although only GP38 protein was shown to be crucial for production of infectious particles, as indicated by total loss of CCHFV tc-VLP infectivity when GP38 is not expressed. A schematic summary of key findings of the present study is given in Fig 7.

### GP38 is critical for envelope glycoproteins maturation and trafficking

The loss in infectivity upon GP38 deletion was found to be associated with reduced levels of Gc maturation and Golgi localization of the latter. In addition, as for preGn in SKI-I/S1P-deficient cells [12], uncleaved preGc was undetectable in the tc-VLP pellets, extending the idea that SKI-I and SKI-I-like processing is required for incorporation of viral glycoproteins and CCHFV infectivity. Accordingly, one possibility is that GP38 expression enhances activity or modulates the substrate specificity of the uncharacterized PC involved in preGc processing. Alternatively, GP38 still fused to MLD may facilitate heterodimer interactions between Gn and Gc, which could be required for proper folding and exposure of preGc PC motif. Indeed, for bunyaviruses, it is generally accepted that Gn/Gc heterodimerization is essential for correct folding and efficient intracellular transport to the Golgi, where viral assembly and budding occur [25]. Likewise, Golgi targeting of CCHFV Gc is dependent on co-expressed Gn [19, 26]. Consistent with this hypothesis, it was previously demonstrated that abrogation of CCHFV Gn N-glycosylation and likely its correct folding resulted in a significant impairment of preGc processing and rendered both Gn and Gc retained in the ER [21]. Interestingly, abrogation of Gn N-glycosylation, leading to ER retention of Gn and Gc, also dramatically affected secretion of GP160, GP85 and GP38 [21]. In this study however, we showed that Gn alone is not capable of directing the transport of Gc to the Golgi implying that for *orthonairoviruses* or at least for CCHFV the interaction between Gn and Gc and formation of hetero-oligomers for proper processing and Golgi transport of Gc is regulated and/or requires GP38.

On this ground, we propose that preGn and preGc assemble into oligomeric structures immediately after SP cleavage events and that these early protein-protein interactions serve important functions for biogenesis of the virus envelope. Indeed, they could mediate the correct folding of the GP precursors with increased PC protease accessibility required for Gn and Gc maturation but also for trafficking of the structural and non-structural secreted proteins along the secretory pathway. This hypothesis is compatible with preGn and preGc cleavage occurring early in the secretory pathway (ER or cis-Golgi) [11] and with the observation that abrogation of SKI-I mediated cleavage of preGn did not impede glycoprotein trafficking to the Golgi apparatus, while it prevented glycoproteins incorporation into particles [12]. However, as previously reported [13], GP160, GP85 and GP38 proteins are well secreted in the absence of Gn expression, indicating that processing and trafficking of the proteins located upstream mature Gn are independent of Gn. It is currently unknown if MLD-GP38 remains associated with CCHFV GP after SKI-I cleavage. Yet, it was recently shown that MLD-GP38/GP38 could be localized to both the plasma membrane and the virus particle envelope [14], which argues in favor of a fraction of MLD-GP38/GP38 remaining associated with CCHFV glycoproteins after assembly and egress.

### GP38 post-ER trafficking is required for efficient Gc incorporation and infectivity

GP38 shares no homology to any other viral or cellular protein and its crystal structure has very recently been solved, revealing a novel fold [27]. However, functionally, GP38 has some similarities to flavivirus nonstructural protein 1 (NS1). Indeed, both proteins are secreted and can attach to cell membranes whereas passive transfer of anti-NS1 [28] and anti-GP38 [14, 27] monoclonal antibodies conferred protection against flaviviruses and CCHFV challenge in small animal models, respectively. Furthermore, NS1 plays a role in virus RNA replication and was recently shown to be crucial for generation of infectious DENV viral particles through its interactions with the structural proteins E and pr-M in the ER lumen [29]. Several studies have shown that similar to CCHFV (this report), flaviviruses mutants lacking NS1 do not replicate but can be rescued by trans-complementation with full length NS1 protein [29, 30].

Assembly of CCHFV particles is a poorly understood process. That MLD-GP38/GP38 are predominantly concentrated in the Golgi, the site of virus assembly and budding, and also co-localize with at least one of the viral structural proteins strongly support the hypothesis of their involvement in CCHFV particle formation. In this report, we showed that MLD-GP38 promotes early vesicular traffic of viral proteins from the sites of CCHFV glycoproteins synthesis, *i*.*e*. ER, to early secretory compartments (ERGIC) and Golgi compartment. Moreover, the assessment that abrogation of GP38 processing from the glycoprotein precursor by mutating the Furin cleavage site and/or deletion of the MLD protein from the M segment polyprotein impacted Gc intracellular distribution, particle assembly and/or GPs incorporation further reinforces this idea. One possible explanation is that MLD protein regulates the accumulation of GPs in the Golgi by slowing down ER exit, resulting in less GPs in the Golgi and decreased production of particles. Accordingly, deletion of the MLD protein resulted in an increased tendency for Gc co-localization in the Golgi and in the formation of particles without altered infectivity but with reduced GPs density. While this last observation might suggest faster GPs trafficking, it could imply that the MLD protein alters GP38 conformation that could impact the subunit stoichiometry of the GPs oligomers in transit in the early secretory pathway.

Here, we devised a tc-VLP-based transcomplementation assay to further investigate the roles of the M-segment encoded accessory proteins in CCHFV assembly and secretion. Using this method, we confirmed that MLD-GP38 is required for traffiicking of viral proteins from the sites of CCHFV glycoproteins synthesis, *to* the Golgi, by trans-complementation with a KDEL-tagged MLD-GP38 (MLD-GP38-KDEL). KDEL-tagged proteins are shuttling between the ER and the Golgi apparatus [20]. Accordingly, we found that MLD-GP38-KDEL could still assist preGc cleavage, but that Gc incorporation was severely impaired as none of the proteins reached the medial Golgi, providing evidence for a previously unreported association between MLD-GP38 and Gc beyond the early secretory compartments (ERGIC) crucial for formation of infectious particles. Similarly, we demonstrated that ectopically expressed MLD-GP38, MLD-GP38 mutant resistant to Furin cleavage, or GP38 lacking the MLD protein could trans-complement the MLD-GP38 double deletion mutant, restoring production and release of infectious tc-VLPs. Consistent with GP38 processing by Furin occurring in TGN, both MLD-GP38/GP38 and Furin-resistant MLD-GP38 mutants supported efficiently preGc to Gc cleavage that takes place early in the secretory pathway and formation of infectious particles. However, neither MLD-GP38 or GP38 in trans-complementation was as efficient as MLD-G38/GP38 resulting from M polyprotein precursor processing in co-localization and targeting Gc to the site of assembly, generally indicating that MLD-GP38/GP38 expression/maturation and their associated functions are more physiological in the context of the M-segment than when expressed ectopically.

### Subversion of the host secretory pathway by NSm promotes CCHFV secretion

Our study also identified NSm as another viral trans-acting factor important for assembly and secretion of CCHFV particles. Deletion of NSm resulted in reduced extra- and intracellular infectious titers that were uncoupled to changes in Gc distribution, which ruled for NSm functions taking place in post-ER compartments. A recent study showed NSm to be dispensable for growth *in vitro* and disease in Ifnar^-/-^ mice using recombinant CCHFV mutant virus lacking the NSm protein [31]. Yet, consistently with our findings, there was a general tendency for the growth kinetics of recombinant CCHFV mutant virus lacking NSm to be slower and the end point titers lower than for NSm expressing CCHFV. *In vivo* analysis also showed that NSm was not required for CCHFV pathology, although animals infected with recombinant CCHFV mutant virus lacking NSm had delayed clinical onset and time of death [31].

NSm expression drastically modulated the N-glycosylation of CCHFV GPs in transit through the secretory pathway. Glycans play important contributions to overall protein structure and functions, and viruses often exploit host-cell machinery to glycosylate their own proteins during replication. This allows to promote protein folding/stability and virion incorporation of GP, protect viral particles from antibody-mediated neutralization, and serve as ligands for virus attachment and entry. That altered glycosylation of CCHFV GPs induced by NSm expression promoted assembly and secretion of infectious particles is surprising and suggests that CCHFV may not require complex type glycans attached on the envelope GPs for virus assembly or for incorporation of these proteins into virus particles. CCHFV M segment expression in HEK293T cells, independently of virus replication, resulted in accumulation of Gn and Gc that were mostly sensitive to endoH treatment [19]. Similarly, Gc from pellets of live CCHFV particles grown on Vero cells remained endoH sensitive while Gn appeared to contain both endoH sensitive and resistant modifications [9], which is compatible with N-glycosylation maturation in the Golgi being a highly complex and cell-type-dependent process [32]. Additionally, it was previously shown that Gc glycosylation is dispensable for processing and trafficking to the Golgi [21], though the impact of glycosylation in assembly and virion secretion was not investigated. Based on the analysis of envelope GPs of different virus origins such as CCHFV, H7N1 and VSV, members of the families Nairoviridae, Orthomyxoviridae, and Rhabdoviridae, respectively, we demonstrate here that NSm expression induces significant changes in the maturation of protein N-glycans, which, most interestingly, did not lead to impaired protein expression or intracellular retention, at least for glycoproteins that assemble at the plasma membrane. Conversely, NSm expression induced a dose-dependent acceleration of a temperature sensitive VSVg mutant, used as a marker of intracellular trafficking, and restored the production and release of infectious tc-VLP.

Acceleration of trafficking through the host secretory pathway as induced, for example, by disruption of the Golgi structure usually results in decreased N-linked glycosylation and N-linked glycan complexity [33]. For bunyaviruses, the signals that initiate virion budding are largely unknown, although a common feature is the accumulation of viral proteins in the Golgi region through the presence of Golgi retention signals in Gn [34–36] that is often associated with expansion of Golgi cisternae and increased vacuolization. Specifically, BUNV-infected cells show tubular structures around the Golgi complex enriched with NSm protein [16]. Partial deletion of NSm first TMD and the cytosolic loop resulted in lower density of tubes, decreased virus yields and intracellular accumulation of immature viral intermediates. Similarly, Oropouche virus (OROV) proteins accumulate in vesicular structures derived from enlarged Golgi cisternae and recruits ESCRT machinery elements to these viral factories derived from the Golgi cisternae [37]. Likewise, the regulation of trafficking rates and GP N-glycosylation by NSm could be an important mechanism evolved by CCHFV to overcome Golgi retention after sufficient accumulation of virus components required for particle formation has occurred and to promote the release of progeny virus. NSm could affect ion concentrations or proteins conformations in order to prevent the action of Golgi enzymes (*N*-acetylglucosaminyltransferase 1 (GlcNAc-T1), galactosyltransferase I (GT1), sialyltransferase I (ST1), mannosidase II (Mann II), *N*-acetylgalactosaminyltransferase (GalNAc-T) 1–3) that may result in a reduced time of retention in Golgi and, thus, an increased speed of secretion. Alternatively, it could generate specific Golgi microenvironment that have a specific composition, *i*.*e*., without Golgi enzymes, and that could lead to a quicker unconventional secretion. Yet, despite the obvious changes in glycosylation, we did not obtain evidence of alterations in the Golgi structure by confocal microscopy as detected for *orthobunyavirus* and as for CCHFV-dead infected embryonic visceral tissue cells [38], and also for other *orthonairoviruses* such DUGV and NSDV, by electron microscopy [39, 40].

In conclusion, we uncovered important but previously unrecognized roles for the nonstructural proteins encoded by the CCHFV M-segment. We demonstrate that GP38 intervenes at early steps of CCHFV GP biogenesis by assisting their processing and trafficking to the Golgi. At the site of CCHFV assembly, presumably through interaction with the virus structural proteins and host factors, GP38 supports assembly of infectious particles. Efficient release of CCHFV requires complex modifications of the host N-glycosylation pathways induced by NSm (Fig 7). Future studies to test our findings in the context of recombinant CCHFV in human and tick cells are warranted. CCHFV is a growing health problem in endemic areas that has been steadily increasing in recent years but also poses new threats as it emerges in new territories. Despite the severity of the disease and the unpredictable nature inherent to zoonotic diseases, there are currently many knowledge gaps in the biology of CCHFV, which in part explain the lack of specific counteracting antiviral therapies and preventive measures. In this study, we identified that the GP38 protein is crucial for formation of infectious particles, while NSm supports their release. Our findings reveal two novel mechanisms that potentially represent virus targets for antiviral therapies.

## Materials and methods

### Cell culture and reagents

Huh-7 hepatocarcinoma and 293T kidney cells were grown in Dulbecco’s modified Eagle’s medium (DMEM) supplemented with 10% fetal calf serum (FCS), and 1% penicillin-streptomycin. All cells were grown in a 37°C and 5% CO_2_ incubator.

### Antibodies

Mouse monoclonal antibodies targeting CCHFV strain Ibar10200 anti-PreGn/GP38 (clone 6B12), anti-PreGn/GP38 (clones 13G8 and 8F10), anti-PreGc (clone 11E7), and anti-NP (clone 9D5) and were obtained from the Joel M. Dalrymple—Clarence J. Peters USAMRIID Antibody Collection through BEI Resources, NIAID, NIH. Rabbit polyclonal anti-Gn antibody has been kindly given by Ali Mirazimi (Karolinska Institute, Sweden). Monoclonal mouse anti-β-actin AC-74 (Sigma), anti-GFP (Roche) and anti-VSV-G 41A1 [41] and rabbit monoclonal anti-GM130 clone EP892Y (Abcam) antibodies were used according to the providers’ instructions.

### Plasmids and constructs

The constructs encoding wild-type CCHFV strain IbAr10200 L polymerase (pCAGGS-V5-L-WT), CCHFV N nucleoprotein (pCAGGS-NP), CCHFV-specific eGFP-expressing minigenome (pT7RiboSM2_vL_eGFP), CCHFV M-segment polyprotein (pCAGGS-GP/wt-M), T7 RNA polymerase (pCAGGS-T7), and an empty vector without viral genes (puc19) were described previously [18, 42]. Several CCHFV M segment cDNA mutants were generated using standard molecular cloning techniques and confirmed by DNA sequencing. Standard PCR and oligonucleotide-specific mutagenesis reactions were carried out with Phusion enzyme (NEBiolabs). Details and oligonucleotides used for the constructs are available upon request. ΔMLD-M, ΔMLDΔGP38-M and ΔMLDΔGP38ΔSKI-M are modified M segments lacking MLD (ΔMLD) or MLD-GP38 (ΔMLDΔGP38). In the first two constructs the N-terminal signal peptide (SP) and the adjacent encoding region (+10 amino acids) were fused to the sequence encoding the last 15 amino acid residues of the MLD protein (aa 232) in ΔMLD-M or the last 13 amino acids of the GP38 protein (aa 506) in ΔMLDΔGP38-M in order to keep the Furin and SKI-I cleavage sites, respectively. In ΔMLDΔGP38ΔSKI-M the SP and the adjacent +2 residues after SP cleavage site were directly fused to the first codon of the Gn protein. Consequently, the N-terminal of Gn is released after signal peptidase mediated cleavage independently of SKI-I processing (Fig 1A). M-ASAA and M-QSQQ are modified M segments encoding a glycoprotein resistant to Furin-like PC endoproteolysis, in which the canonical RSKR motif was changed to ASAA or QSQQ, respectively. Wt-M, ΔMLD-M, M-ASAA and M-QSQQ were used as templates for PCR amplification of MLD, MLD-GP38, GP38 only or MLD-GP38 resistant to Furin cleavage (MLD-GP38ASAA/QSQQ) encoding regions that after were subcloned into the plasmid pCAGGS. MLD-GP38-KDEL was created by mutagenesis of the last amino acid of the SKI-I/S1P cleavage site (RRLL to RRLA) and fusion to a KDEL motif. By introducing a stop codon at different positions of the CCHFV M segment four C-terminal truncation mutants were created. They encode either for the full-length preGn polyprotein (preGn999) ending at position 999, +4 residues downstream the internal SP cleavage site of preGc or smaller preGn variants (preGn961, preGn881 and preGn856) lacking gradually larger regions of NSm protein ending at amino acid positions 961, 881 or 856 of the M-segment. SP-preGc construct encodes the precursor preGc in addition to the 40 amino acid sequence located upstream the internal SP cleavage site between NSm and preGc. To generate NSm construct a stop codon was introduced at position 1000 of CCHFV M-polyprotein in a plasmid pCGGS harboring NSm and Gc sequence (sequence encoding for amino acids 808 till the end of CCHFV M polyprotein).

The plasmid pEGFP-N3-VSV-Gts was a kind gift from Konan Kouacou (Albany Medical College, USA).

### Production of tc-VLPs

Huh7 cells were seeded in 6-well plates and transfected with 600 ng of pCAGGS-V5-L WT, 200 ng of pCAGGS-N, 200 ng of pT7riboSM2-vS-GFP, 500 ng of pCAGGS-GP, 500 ng of pCAGGS-T7, using GeneJammer transfection reagent (Agilent). Puc19 plasmid was additionally transfected to keep the DNA amount uniform. The transfection media was replaced after 6h post transfection. Cells supernatants were harvested 72h post transfection, filtered through a 0.45 μm filter, and concentrated by ultrafiltration (Vivaspin 100MWCO, Sartorius) or by ultracentrifugation through a 20% sucrose cushion (SW41 rotor at 28,000 rpm, 4h, 4°C).

### Intracellular and extracellular infectivity

Infectivity was analyzed by infecting naïve L and N pre-transfected Huh7 cells. Briefly, Huh7 cells were seeded in 24-well plates and pre-transfected with 100 ng pCAGGS-V5-L-WT and 200 ng pCAGGS-N, prior to infection. Intracellular tc-VLP particles were released by three repeated freeze-thaw cycles. The infectivity was analyzed 24h post-infection by flow cytometry, using a MACSQuant VYB apparatus (Miltenyi Biotec). Data were then analyzed using the FlowJo software. Titers were calculated according to the formula (number of seeded Huh7 cells x % of GFP-positive cells) x 1000/ μl of inoculum and expressed as infectious units per ml of supernatant (extracellular infectivity) or cell lysate (intracellular infectivity).

### Deglycosylation with endoglycosidase Hf and PNGase

Endoglycosidase Hf (Endo-Hf, NEBiolabs) and Peptide-N-Glycosidase F (PNGase F, NEBiolabs) treatments were performed according to the manufacturer’s recommendations. Briefly, cells lysates and pellets samples were mixed to denaturing glycoprotein buffer and heated at 95°C for 5 min. Then, Endo-Hf (1,000 units) or PNGase F (500 unit) were added to samples in a final volume of 25 μl, and placed at 37°C for 2h, before western blot analysis.

### Western blot analysis

Transfected cells were washed with cold phosphate-buffered saline (PBS) and lysed in cold cell lysate buffer (20 mM Tris pH 7.5, 1% Triton, SDS 0.05%, Na Deoxycholate Acid 0.5% and 150 mM NaCl) containing protease inhibitors (Roche). Nuclei and membranes were precipitated by centrifugation at 12,000 rpm for 20 min. Pellets from ultracentrifugated supernatants were resuspended in PBS 1x buffer in 1/100 of the initial volumes.

For detection of Gn, samples were denaturated at 95°C for 5 min, in reducing loading buffer (5X Blue Loading Buffer, 200 mM Tris HCl pH6.8, 10% SDS, 500 mM β-mercaptoethanol and 50% glycerol) and electrophoresed on 14% SDS-polyacrylamide gels. Alternatively, for detection of Gc, samples were processed in non-reducing loading buffer (5X Blue Loading Buffer, 200 mM Tris HCl pH6.8, 20% SDS, and 50% glycerol) and electrophoresed on 9% polyacrylamide gels. Proteins were transferred onto a nitrocellulose membrane by electroblotting (BioRad) for 1h at 100V. After saturation in TBST (20 mM Tris HCl, pH 7.5, 150 mM NaCl and Tween 0.05%)-milk 5% for 1h, membranes were incubated with primary antibodies for the detection of CCHFV proteins (mouse monoclonal 11E7 anti-Gc 1:500, rabbit polyclonal anti-Gn 1:5,000 and mouse monoclonal 9D5 anti-NP 1:1,000), in TBST-milk 5% overnight at 4°C. After 3 washes of 10 min using TBST, membranes were then incubated with IRdye secondary antibodies (Li-Cor Biosciences) at 1:10,000 dilution in TBST for 1h at room temperature, followed by imaging with Odyssey infrared imaging CLx system (Li-Cor Biosciences). Quantification of proteins was performed with Odyssey imaging CLx system software.

### Immunofluorescence analysis

Briefly, Huh7 cells were seeded in 24-well plates on coverslips and transfected with the plasmids described above. The transfection media was replaced after 6h post transfection and, 48h after transfection, the cells were fixed with 4% paraformaldehyde for 15 min at room temperature, then permeabilized with 0.1% Triton X-100 for 3 min. Cells were washed with PBS and incubated for 1 h at room temperature with primary antibodies, followed by incubation with a fluorophore-conjugated secondary antibody in 1%BSA/PBS. For some experiments, anti-desmin antibody (AbCam) was used together with a different fluorophore-conjugated secondary antibody (Jackson Laboratories). Nuclei were stained with Hoechst (Molecular Probes) and the coverslips were mounted with Mowiol 4–88 (Sigma-Aldrich). The slides were examined using a LSM-800 (Zeiss) confocal microscope. Pearson’s correlation coefficients were calculated using FIJI (JACoP) were calculated on 15 cells from 3 separated experiments and expressed as mean (and SEM).

### Production of H7N1 pseudoparticles

H7N1 pseudotyped particles were designed and generated as previously described [43]. Briefly, 293T cells were cultured in 10-cm plates and transfected by calcium phosphate with three expression plasmids encoding (i) murine leukemia virus (MLV) core-packaging components (8 μg); (ii) an MLV retroviral transfer vector harboring the green fluorescent protein (GFP) marker (8 μg); (iii) the H7N1 influenza envelope glycoproteins (HA 2.7 μg, NA 4 μg) to generate the pseudoparticles. Plasmid pCAGGS-NSm (500 ng) encoding putative modulators of intracellular trafficking or glycosylation was added to the transfection mixture. Control pseudoparticles generated with no envelope glycoprotein (No-GP) were generated by transfecting expression plasmids for GFP, MLV core-packaging components and puc19. Cell supernatants were recovered at 36h post transfection, filtered through a 0.45μm filter and concentrated by ultracentrifugation through a 20% sucrose cushion at 25,000 rpm for 1.5h and 4°C (SW41 rotor). Cell lysates and viral pellets were then used in Western blot analysis using a rabbit anti-HA7 (kpRo, 1:1,000) antibody (kindly provided by W. Garten, Marburg, Germany) and a rat anti-MLV-CA (R187 ATCC-CRL1912; 1:1,000) antibody, as above described.

### VSV-G thermo-sensitive analysis

Huh7 cells were seeded 16h prior to transfection with pEGFP-N3-VSV-Gts and NSm-encoding plasmid or phCMV- ΔE2p7 (JFH1) as control, using GeneJammer transfection reagent (Agilent). Medium was changed 4h post-transfection and cells were incubated overnight at 40°C. 24h post-transfection, cells were chased at 32°C. For western blot analysis, cells were lysed at indicated time points in wells cooled on ice before clarification, endoHf treatment and western blot analysis with anti-GFP antibody. For flow cytometry analysis, cells were harvested and put in suspension at 32°C. At indicated time points, cells were fixed with 3% paraformaldehyde. Cell surface staining of VSV-Gts was assessed using the 41A1 mAb directed against VSV-G ectodomain and anti-mouse APC.

### Statistical analysis

Statistical analyses were performed using GraphPad Prism version 5.02 for Windows, GraphPad Software (San Diego California, USA). The Mann-Whitney or Wilcoxon tests were used for statistical comparisons. A p-value of 0.05 or less was considered as significant. When applicable, data are presented as mean ± standard deviation and results of the statistical analysis are shown as follows: ns, not significant (P > 0.05); *, P < 0.05; **, P < 0.01; and ***, P < 0.001.

## Supporting information

S1 Fig CCHFV infectious and non-infectious subviral particles. Western blot analysis of infectious tc-VLPs, glycoprotein deficient NP-containing subviral particles and subviral particles without “capsid” or genome. Lysates (**A**) and ultracentrifuged supernatants through sucrose cushion (**B**) of non-transfected Huh7 cells (lane 1) or Huh7 cells transfected with all tc-VLP assembly plasmids (lane 3), all tc-VLPs plasmids without wt-M (lane 2) or cells transfected with the wt-M construct only (lane 4) were analyzed by western blotting using antibodies against NP, Gc, Gn and actin (lysates only).
(TIF)

S2 Fig Monoclonal anti-preGn antibodies exclusively target GP38 protein. Confocal microscopy analysis of Huh7 cells transfected with pUC19-empty vector, wt-M, ΔMLD-M, ΔMLDΔGP38ΔSKI-1-M, MLD-GP38, MLD-GP38-HA, GP38. At 48h post-transfection, cells were fixed, permeabilized with Triton X-100, and stained for GP38 (6B12, 8F10) or HA (Green channel), Golgi (anti-GM130, blue channel) and nuclei (Hoechst, grey channel).
(TIF)

S3 Fig Intracellular distribution of CCHFV Gc and GP38 over time. Confocal microscopy analysis of Huh7 cells transfected with wt-M. At different time post-transfection, cells were fixed, permeabilized with Triton X-100, and stained for Gc (11E7, red channel), Golgi (anti-GM130, blue channel) and nuclei (Hoechst, grey channel) (**A**) or for GP38 (6B12, green channel), Golgi (anti-GM130, blue channel) and nuclei (Hoechst, grey channel) (**B**).
(TIF)

S4 Fig GP85/GP38 dose-dependent increase of intracellular preGc to Gc conversion, Gc incorporation into particles and infectivity. CCHFV tc-VLPs were generated by co-transfection of ΔMLDΔGP38ΔSKI-M deletion mutant and pUC19 or increasing amounts (250, 125 and 62.5 ηg plasmid DNA) of MLD-GP38 or GP38 only. Infectivity, CCHFV protein expression and particles were analyzed at 72h post-transfection. Western blot analysis using anti-Gc antibody and relative quantification of mature to total Gc ratio in lysates (**A**), mature Gc incorporation into particles (**C**), expressed as fold change compared to pUC19. Infectious tc-VLP titers were determined by FACS 24h post-inoculation of clarified crude supernatants on L and N pre-transfected cells (**B**). Concentrated supernatants by filtration were blotted with anti-GP38 antibody. Note that, GP38 derived from GP38-only construct appears to be more efficiently secreted (**D**).
(TIF)

S5 Fig MLD-GP38 secretion is mostly impaired when fused to a KDEL motif. Western blot analysis of tc-VLPs produced by trans-complementation of the double deletion mutant with MLD-GP38, MLD-GP38HA and MLD-GP38-HA-KDEL. Huh7 cells were co-transfected with the tc-VLP assembly plasmids including a 1.1 mixture of ΔMLDΔGP38ΔSKI-M and pUC19, MLD-GP38, MLD-GP38-HA or MLD-G38-HA-KDEL. Cells lysates and ultracentrifuged supernatants (pellets) were blotted with anti-Gc and Gc antibodies. Supernatants concentrated by filtration were blotted with anti-GP38 and anti-NP antibodies. While NP was detected in all supernatants, MLD-GP38 and GP38 proteins were only detected in the supernatants of tc-VLP-producer cells co-expressing MLD-GP38 and MLD-GP38-HA but not from MLD-G38-HA-KDEL transfected cells.
(TIF)

S6 Fig GP85 to GP38 processing enhances CCHFV tc-VLP production. (**A**) Schematic representation of wt-M, M-ASAA and M-QSQQ (M segments in which the RSKR^247^ Furin cleavage motif has been mutated to either ASAA or QSQQ), ΔMLDΔGP38ΔSKI-M segment, MLD-GP38, and MLD-GP38-ASAA/QSQQ expressing constructs. CCHFV tc-VLPs were generated using constructs encoding either wt-M polyprotein, M-ASAA, and M-QSQQ or by trans-complementation of the ΔMLDΔGP38ΔSKI-M deletion mutant with either pUC19, or with MLD-GP38, MLD-GP38-ASAA or MLD-GP38-QSQQ expression vectors. Infectivity, CCHFV protein expression and tc-VLP secretion were analyzed at 72h post-transfection. (**B**) Clarified supernatants were inoculated on L and N pre-transfected Huh7 cells and infectious titers were determined by FACS 24h post-infection. (**C-E**) Intracellular levels of CCHFV proteins expression and processing. Cell lysates of tc-VLP-producing cells were analyzed by Western blotting with antibodies against the indicated proteins including Gn, Gc, NP and host actin. Intracellular protein band intensities were quantified and normalized relative to actin and expressed as fold change compared to wt-M. Representative western blot analysis and relative quantification of intracellular Gn (**C**), preGc and Gc (**D**), and NP (**E**) protein levels. (**F-H**) tc-VLP secretion. Western blot analysis of tc-VLP-associated proteins purified by ultracentrifugation through 20% sucrose cushion. Representative blot analysis of Gn (**F**), Gc (**G**) and NP (**H**) expressed as fold change relative to wt-M. (**I-J**) Western blot analysis of cell supernatants concentrated by ultrafiltration blotted with anti-GP38 antibody. Molecular weight markers are marked on the left.
(TIF)

S7 Fig RSKR^247^ Furin cleavage mutation does not modify GP38 and Gc protein (co)localization. (**A)** Confocal microscopy analysis of Huh7 cells transfected with different expression plasmids as indicated. At 48h post-transfection, cells were fixed, permeabilized with Triton X-100, and stained for GP38 (6B12, green channel), Gc (11E7, red channel), Golgi (anti-GM130, cyan channel) and nuclei (Hoechst, grey channel). (**B**) Pearson’s coefficients were calculated using FIJI (JACoP) on 25 cells and expressed as mean (and SEM). Scale bars represent 10μm.
(TIF)
