# Supplementary figures and images for: The interplays between Crimean-Congo hemorrhagic fever virus (CCHFV) M segment-encoded accessory proteins and structural proteins promote virus assembly and infectivity
Source: PLoS Pathog. 2020 Sep 21;16(9):e1008850. doi: 10.1371/journal.ppat.1008850 (PMC7529341; doi:10.1371/journal.ppat.1008850)

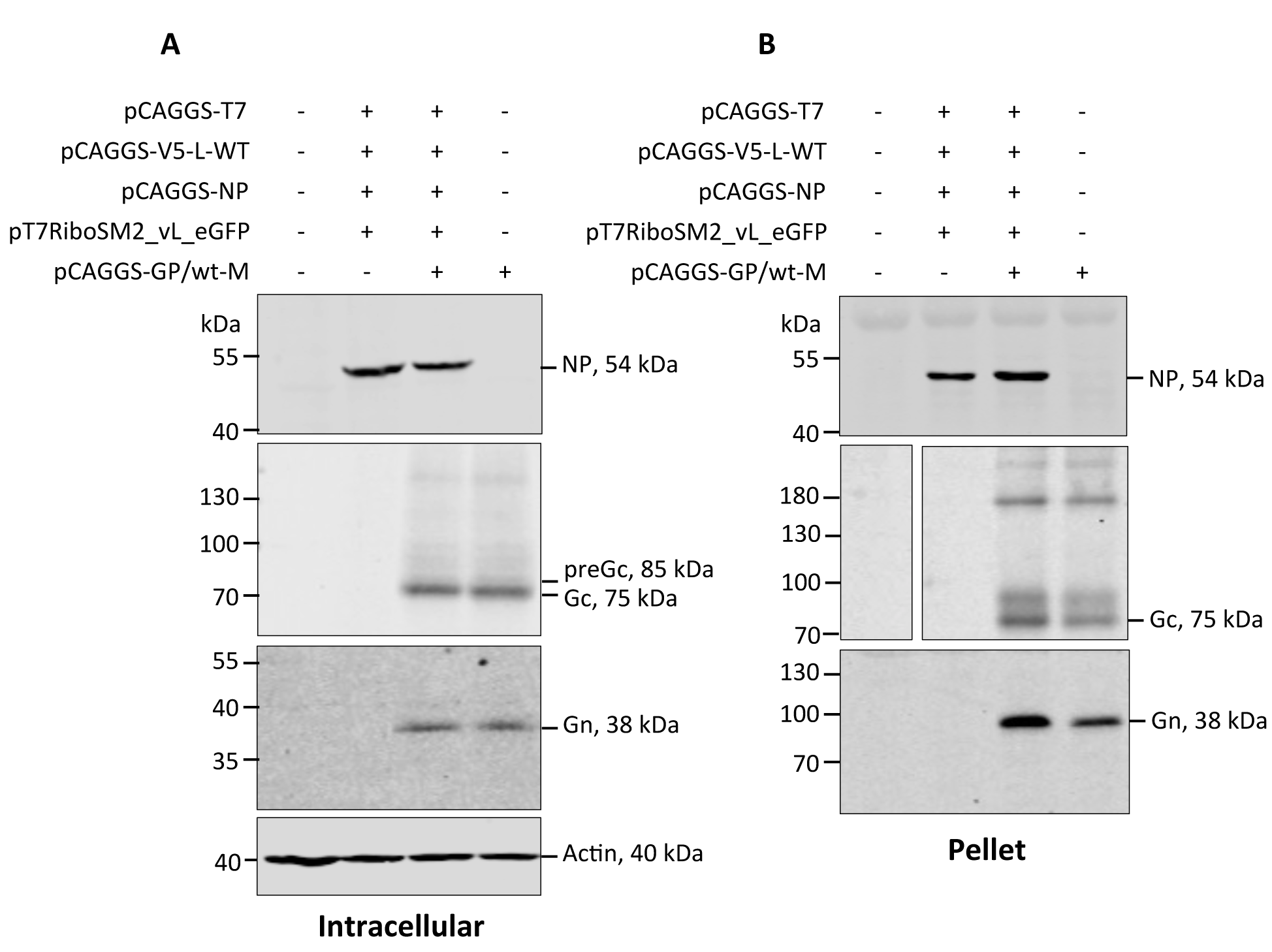

Supplement: S1 Fig — Western blot analysis of infectious tc-VLPs, glycoprotein deficient NP-containing subviral particles and subviral particles without “capsid” or genome. Lysates (A) and ultracentrifuged supernatants through sucrose cushion (B) of non-transfected Huh7 cells (lane 1) or Huh7 cells transfected with all tc-VLP assembly plasmids (lane 3), all tc-VLPs plasmids without wt-M (lane 2) or cells transfected with the wt-M construct only (lane 4) were analyzed by western blotting using antibodies against NP, Gc, Gn and actin (lysates only). (TIF) [file ppat.1008850.s001.tif]

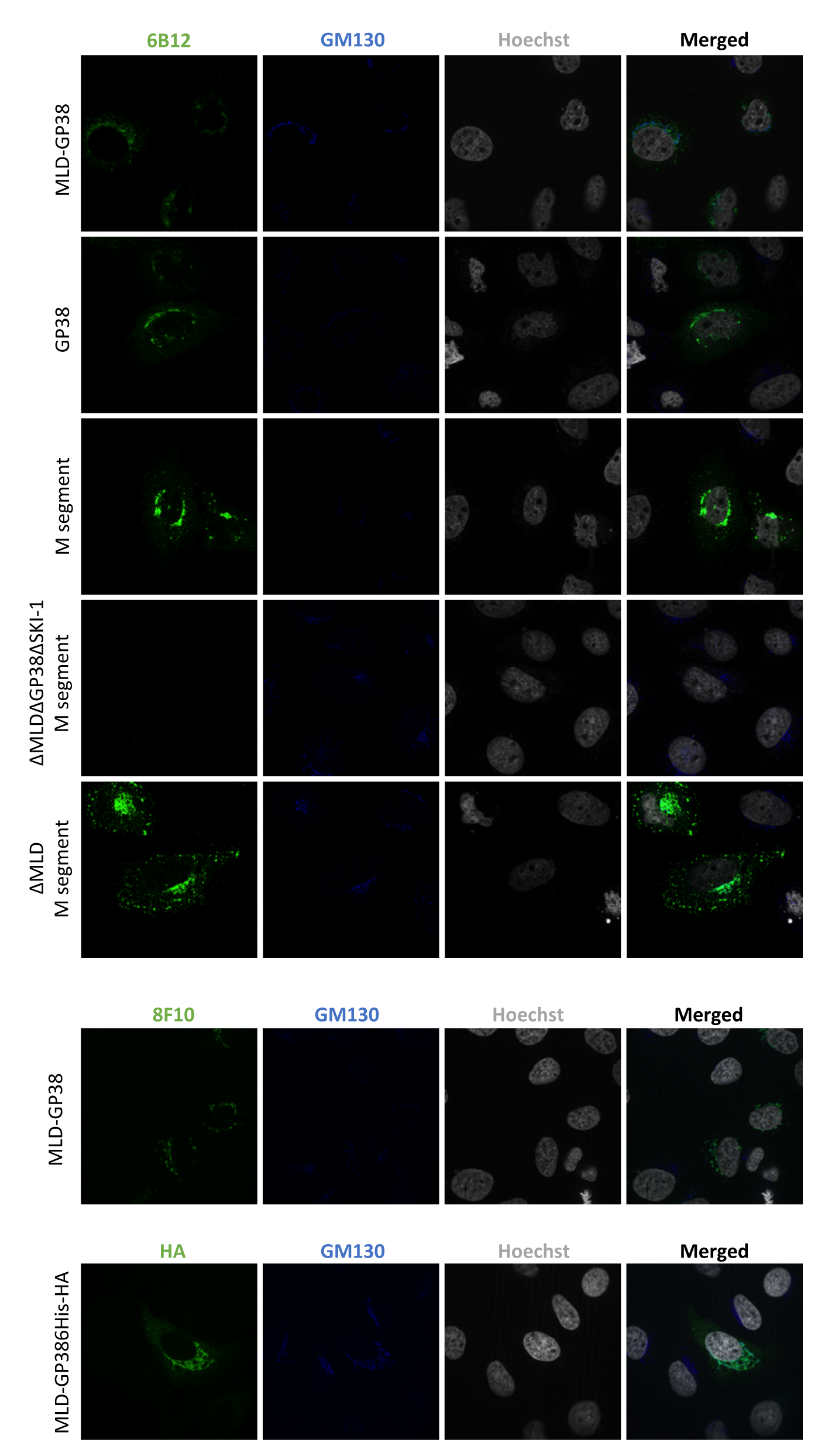

Supplement: S2 Fig — Confocal microscopy analysis of Huh7 cells transfected with pUC19-empty vector, wt-M, ΔMLD-M, ΔMLDΔGP38ΔSKI-1-M, MLD-GP38, MLD-GP38-HA, GP38. At 48h post-transfection, cells were fixed, permeabilized with Triton X-100, and stained for GP38 (6B12, 8F10) or HA (Green channel), Golgi (anti-GM130, blue channel) and nuclei (Hoechst, grey channel). (TIF) [file ppat.1008850.s002.tif]

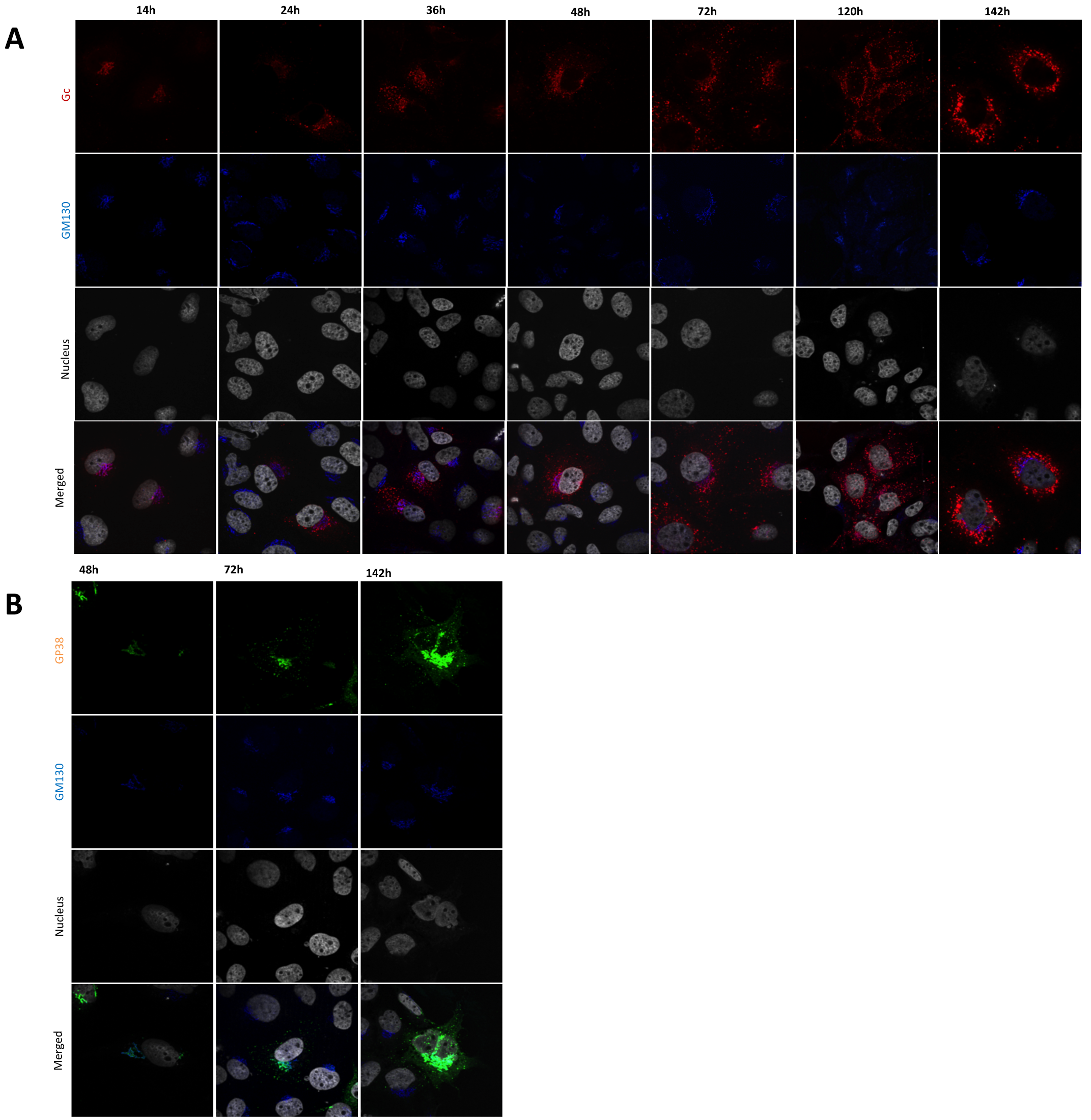

Supplement: S3 Fig — Confocal microscopy analysis of Huh7 cells transfected with wt-M. At different time post-transfection, cells were fixed, permeabilized with Triton X-100, and stained for Gc (11E7, red channel), Golgi (anti-GM130, blue channel) and nuclei (Hoechst, grey channel) (A) or for GP38 (6B12, green channel), Golgi (anti-GM130, blue channel) and nuclei (Hoechst, grey channel) (B). (TIF) [file ppat.1008850.s003.tif]

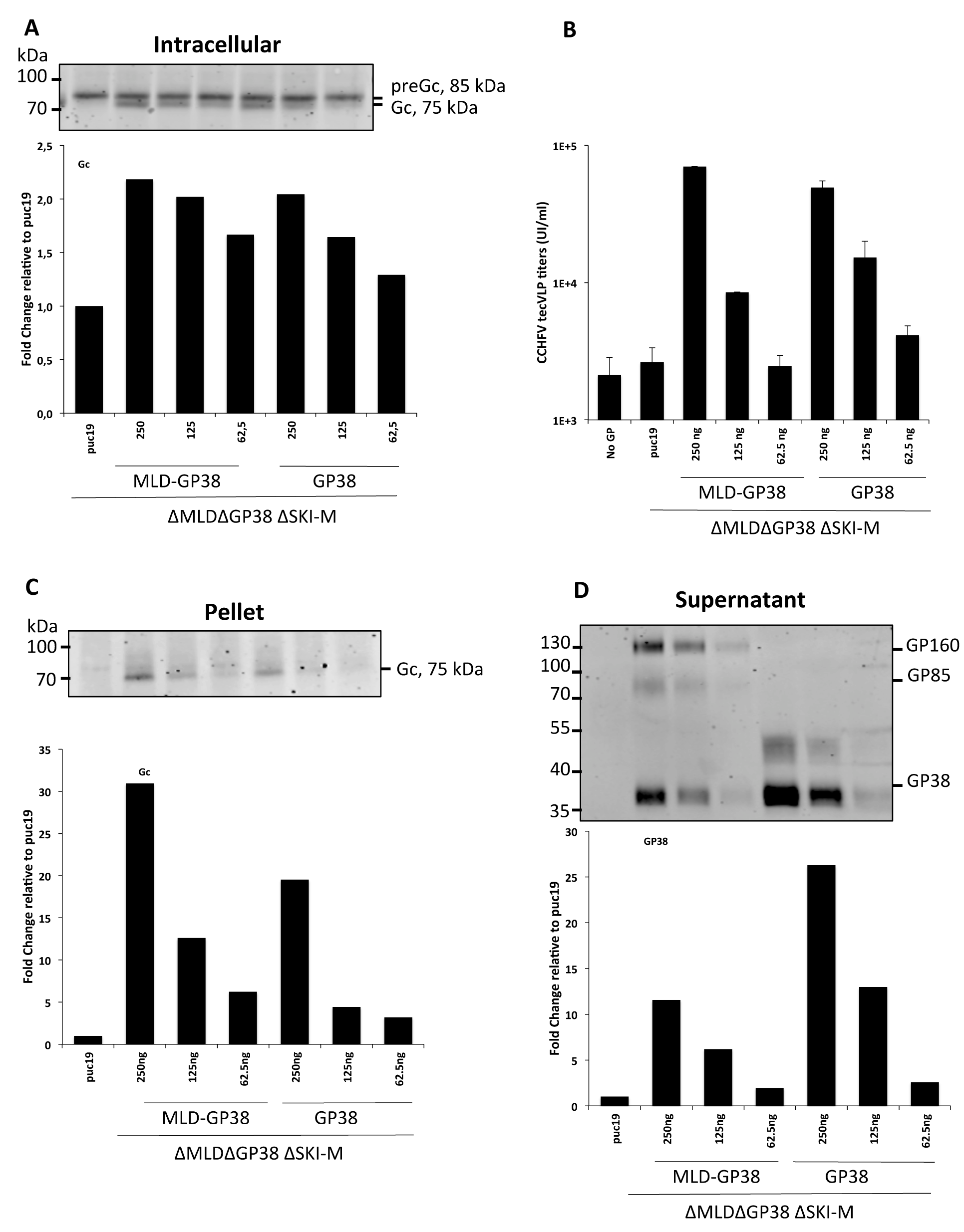

Supplement: S4 Fig — CCHFV tc-VLPs were generated by co-transfection of ΔMLDΔGP38ΔSKI-M deletion mutant and pUC19 or increasing amounts (250, 125 and 62.5 ηg plasmid DNA) of MLD-GP38 or GP38 only. Infectivity, CCHFV protein expression and particles were analyzed at 72h post-transfection. Western blot analysis using anti-Gc antibody and relative quantification of mature to total Gc ratio in lysates (A), mature Gc incorporation into particles (C), expressed as fold change compared to pUC19. Infectious tc-VLP titers were determined by FACS 24h post-inoculation of clarified crude supernatants on L and N pre-transfected cells (B). Concentrated supernatants by filtration were blotted with anti-GP38 antibody. Note that, GP38 derived from GP38-only construct appears to be more efficiently secreted (D). (TIF) [file ppat.1008850.s004.tif]

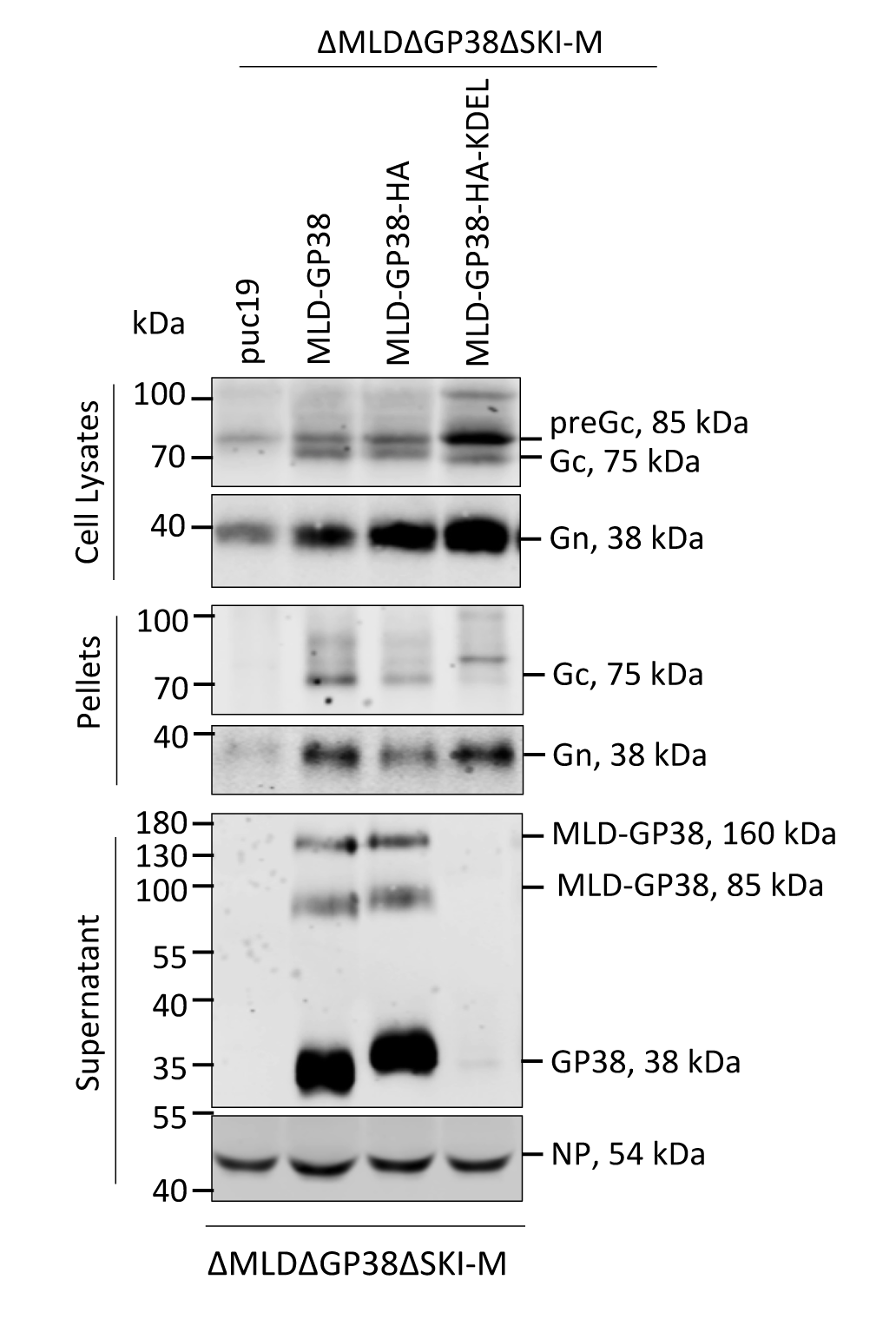

Supplement: S5 Fig — Western blot analysis of tc-VLPs produced by trans-complementation of the double deletion mutant with MLD-GP38, MLD-GP38HA and MLD-GP38-HA-KDEL. Huh7 cells were co-transfected with the tc-VLP assembly plasmids including a 1.1 mixture of ΔMLDΔGP38ΔSKI-M and pUC19, MLD-GP38, MLD-GP38-HA or MLD-G38-HA-KDEL. Cells lysates and ultracentrifuged supernatants (pellets) were blotted with anti-Gc and Gc antibodies. Supernatants concentrated by filtration were blotted with anti-GP38 and anti-NP antibodies. While NP was detected in all supernatants, MLD-GP38 and GP38 proteins were only detected in the supernatants of tc-VLP-producer cells co-expressing MLD-GP38 and MLD-GP38-HA but not from MLD-G38-HA-KDEL transfected cells. (TIF) [file ppat.1008850.s005.tif]

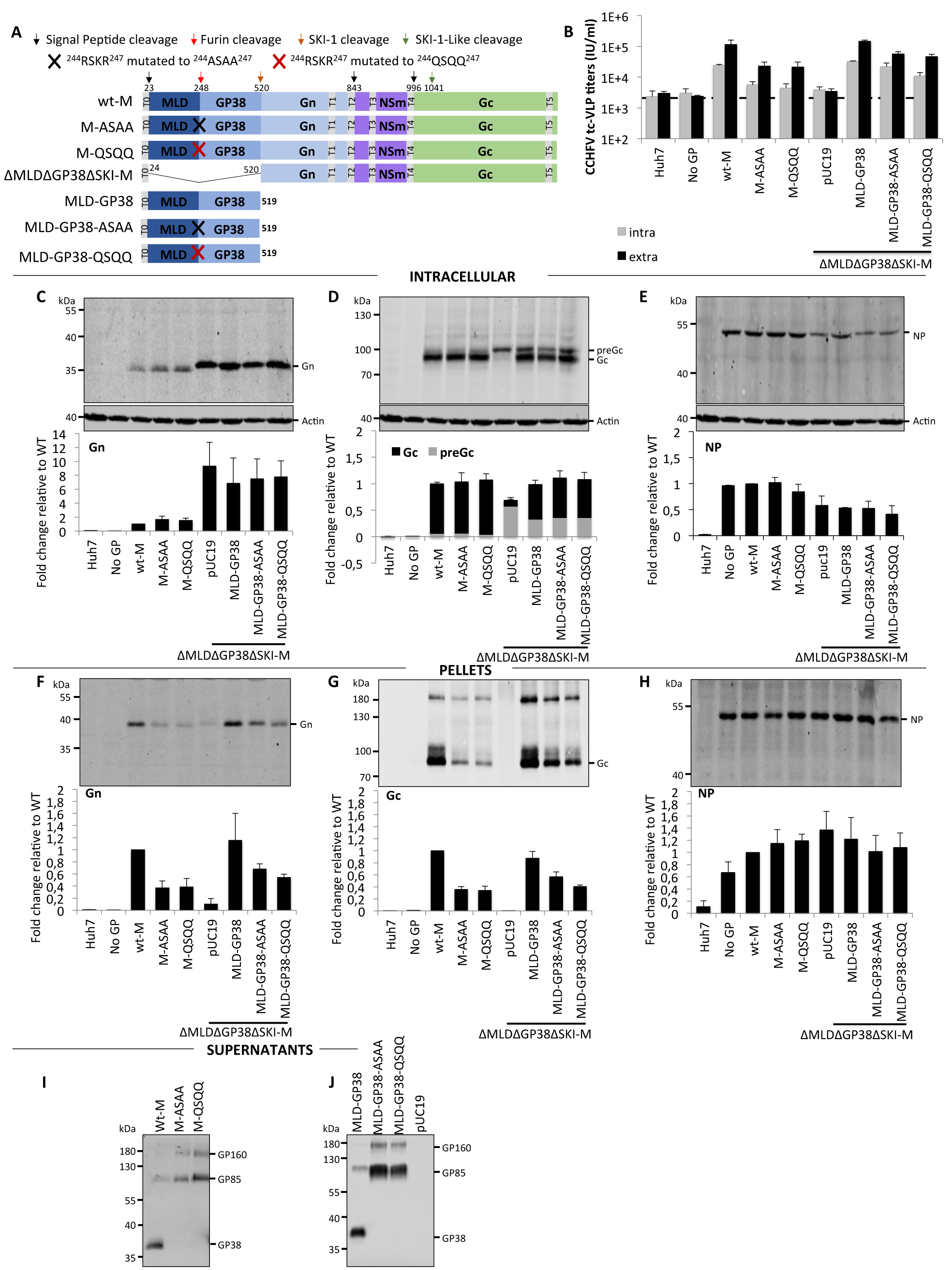

Supplement: S6 Fig — (A) Schematic representation of wt-M, M-ASAA and M-QSQQ (M segments in which the RSKR247 Furin cleavage motif has been mutated to either ASAA or QSQQ), ΔMLDΔGP38ΔSKI-M segment, MLD-GP38, and MLD-GP38-ASAA/QSQQ expressing constructs. CCHFV tc-VLPs were generated using constructs encoding either wt-M polyprotein, M-ASAA, and M-QSQQ or by trans-complementation of the ΔMLDΔGP38ΔSKI-M deletion mutant with either pUC19, or with MLD-GP38, MLD-GP38-ASAA or MLD-GP38-QSQQ expression vectors. Infectivity, CCHFV protein expression and tc-VLP secretion were analyzed at 72h post-transfection. (B) Clarified supernatants were inoculated on L and N pre-transfected Huh7 cells and infectious titers were determined by FACS 24h post-infection. (C-E) Intracellular levels of CCHFV proteins expression and processing. Cell lysates of tc-VLP-producing cells were analyzed by Western blotting with antibodies against the indicated proteins including Gn, Gc, NP and host actin. Intracellular protein band intensities were quantified and normalized relative to actin and expressed as fold change compared to wt-M. Representative western blot analysis and relative quantification of intracellular Gn (C), preGc and Gc (D), and NP (E) protein levels. (F-H) tc-VLP secretion. Western blot analysis of tc-VLP-associated proteins purified by ultracentrifugation through 20% sucrose cushion. Representative blot analysis of Gn (F), Gc (G) and NP (H) expressed as fold change relative to wt-M. (I-J) Western blot analysis of cell supernatants concentrated by ultrafiltration blotted with anti-GP38 antibody. Molecular weight markers are marked on the left. (TIF) [file ppat.1008850.s006.tif]

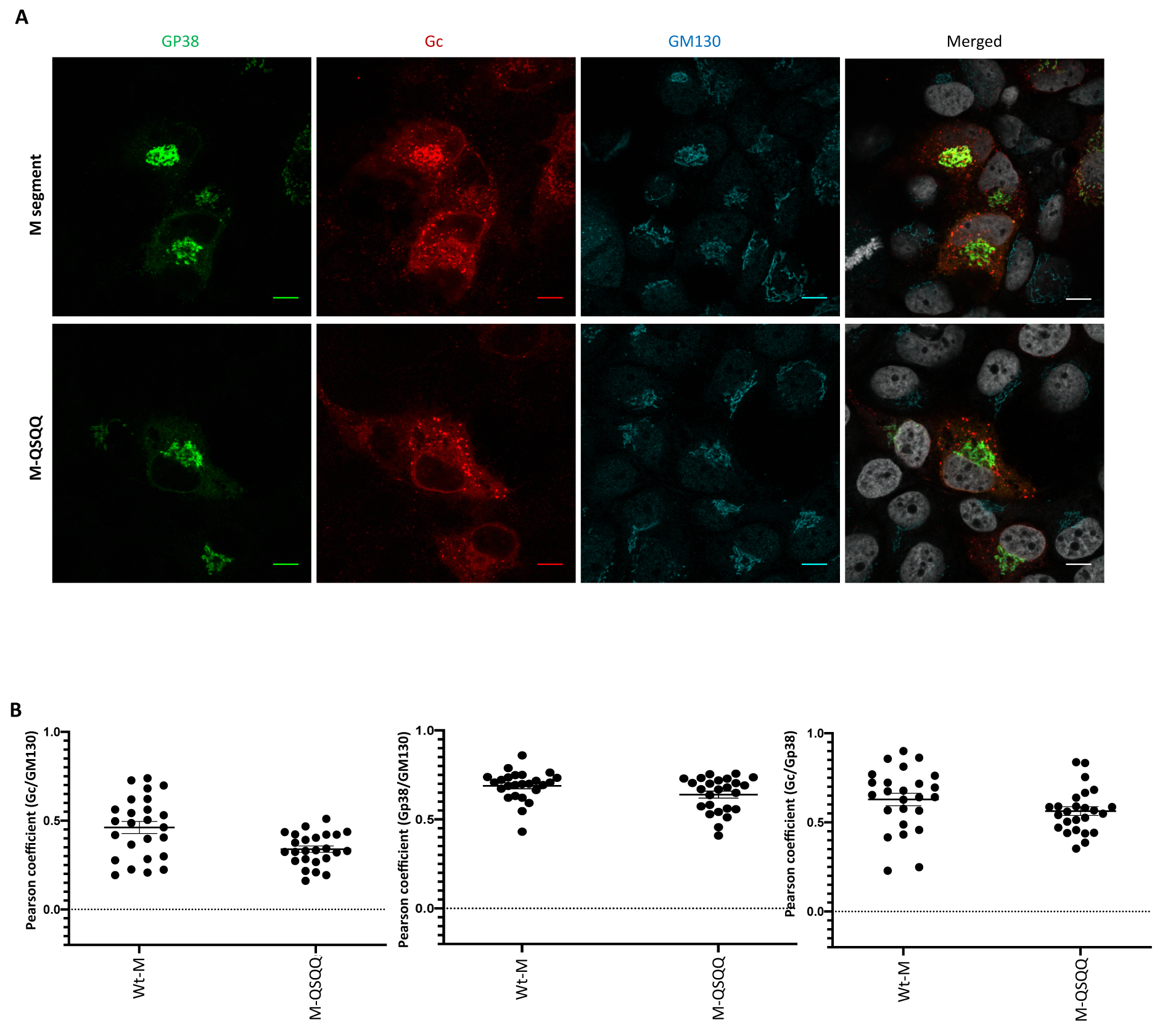

Supplement: S7 Fig — (A) Confocal microscopy analysis of Huh7 cells transfected with different expression plasmids as indicated. At 48h post-transfection, cells were fixed, permeabilized with Triton X-100, and stained for GP38 (6B12, green channel), Gc (11E7, red channel), Golgi (anti-GM130, cyan channel) and nuclei (Hoechst, grey channel). (B) Pearson’s coefficients were calculated using FIJI (JACoP) on 25 cells and expressed as mean (and SEM). Scale bars represent 10μm. (TIF) [file ppat.1008850.s007.tif]
